# Microglia surveillance is directed toward neuron activation during sustained intracortical microstimulation

**DOI:** 10.1088/1741-2552/ae4652

**Published:** 2026-02-27

**Authors:** Colin Preszler, Kevin C Stieger, Keying Chen, Guangfeng Zhang, Takashi D Y Kozai

**Affiliations:** 1 Department of Bioengineering, University of Pittsburgh, Pittsburgh, PA, United States of America; 2 Center for the Neural Basis of Cognition, Pittsburgh, PA, United States of America; 3 Center for Neuroscience, University of Pittsburgh, Pittsburgh, PA, United States of America; 4 McGowan Institute for Regenerative Medicine, University of Pittsburgh, Pittsburgh, PA, United States of America; 5 Neuroscience Institute, Carnegie Mellon University, Pittsburgh, PA, United States of America

**Keywords:** gliomodulation, immunoelectrophysiology, antidromic activation, gliosynaptic transmission, microglia–neuron crosstalk, neural hysteresis, neuroimmune feedback

## Abstract

*Objective.* Intracortical microstimulation (ICMS) is a widely used tool for neuroprostheses, but its long-term efficacy is often limited by foreign body response and neuroinflammatory responses at the electrode-tissue interface. Microglia orchestrate neuroinflammation and regulate synaptic plasticity, and low-frequency stimulation has been shown to promote anti-inflammatory microglial phenotypes. *Approach.* We investigated how 10 Hz ICMS influences microglia–neuron interactions during the first three days post-implantation using *in vivo* two-photon imaging in Cx3cr1-GFP/jRGECO1a mice. Microglial motility, morphology, and process orientation were tracked relative to electrode placement and neuronal calcium activity (measured as change in fluorescence, Δ*F*/*F*). *Main results.* A 1 h session of 10 Hz ICMS did not induce overt classical morphological activation of microglia but robustly increased process motility, with extensions dynamically tracking neurons showing early activation or subsequent functional suppression. By post-implantation Day 2, microglial processes were significantly more likely to engage neurons with high early calcium responses after stimulation onset (mean orientation angle: 74.3° ± 11.8°), but this engagement shifted during prolonged stimulation (117.0° ± 9.2°, *p* = 0.0017), indicating context-dependent interactions. Contact frequency scaled with neuronal adaptation profiles, and neurons exhibiting depressed activity received the most contacts immediately after implantation (1.2 ± 0.3 contacts, *p* = 0.046). *Significance.* These findings reveal stimulus-associated, neuron-dependent surveillance behaviors of microglia during early post-implantation ICMS and suggest that microglia actively participate in short-term modulation of stimulated cortical circuits.

## 1. Introduction

Electrical stimulation is a cornerstone of neurotechnology, enabling therapeutic neuromodulation and bidirectional communication with neural circuits via implanted devices [1–4]. However, long-term efficacy is often limited by foreign body response and chronic neuroinflammatory responses triggered by electrode-tissue interfaces [5–7]. While neuronal responses to intracortical microstimulation (ICMS) are well-characterized [8–12], the concurrent effects of electrical stimulation on microglia [13, 14], the brain’s primary immune cells [15–17], and subsequent ICMS outcomes remain poorly understood [18, 19].

Microglia are not merely passive responders to injury, they dynamically regulate synaptic plasticity, neuroinflammation, and electrode biocompatibility through cytokine signaling and the formation of physical barriers in brain tissue [19, 20]. Electrical stimulation through conductive materials may influence these microglia–neuron interactions [21], thereby potentially modulating glia-neuron signaling and impacting the fidelity and longevity of neuromodulation. Although microglia do not generate action potentials, they can express voltage-sensitive receptors, and electrochemically sensitive pathways [5, 6, 22]. Electric fields can perturb extracellular ion concentrations (e.g. Ca^2+^, K^+^) or redistribute charge across the microglial membrane, activating downstream pathways that mediate pro-inflammatory cytokine release or anti-inflammatory signaling [23–25]. Notably, *in vitro* studies demonstrate that microglia exhibit directed migration, increased process motility, and morphological remodeling in response to direct electric fields [14, 18]. However, whether microglia respond to ongoing stimulation *in vivo* and how this influences their interactions with neurons remain unresolved.

Following device implantation, microglia transition to a reactive state within minutes by extending processes toward the implant site[26] followed by the release of pro-inflammatory cytokines within hours [22, 27, 28]. These cytokines exacerbate oxidative stress and neuronal damage while also driving glial scar formation[29], which increases electrochemical impedance [30–32] and degrades signal fidelity [33–35]. However, microglia also clear cellular debris[36] and secrete neurotrophic factors [16, 37] indicating a context-dependent role that could be harnessed to improve device integration. For example, fractalkine signaling (CX3CL1–CX3CR1) downregulates excessive microglial reactivity [27, 38], while adenosine (a byproduct of microglial ATP metabolism) suppresses neuronal hyperexcitability [24, 39–41]. Low-frequency ICMS (2–20 Hz) has been shown to bias microglia toward pro-regenerative phenotypes, increasing trophic factor release and reducing inflammatory cytokines [42, 43]. In neuroprosthetic applications, this suggests a strategy wherein appropriately tuned electrical stimulation could modulate microglial states to attenuate cytokine-driven inflammation to promote microglia-mediated tissue integration at the electrode interface.

Here, we investigate how 1 h of 10 Hz ICMS influences microglial-neuron interactions *in vivo* using two-photon imaging in mice expressing GFP in microglia and jRGECO1a in Thy1+ neurons (Cx3CR1-GFP × Thy1-jRGECO1a.GP8.62), which label most excitatory neurons in visual cortex [44]. We selected 10 Hz because it lies within the biologically relevant low-frequency range, has been consistently shown to bias microglia toward pro-regenerative states [42, 43]. However, its effect on dynamic microglial morphological activity remains unclear. Additionally, since microglia have been demonstrated to suppress neural activity [45, 46], we asked whether stimulation alters microglial process dynamics and whether these interactions depend on the activity state of nearby neurons. Our findings show that ICMS induces microglial process extension toward both the electrode and activated neurons, with microglia preferentially contacting neurons exhibiting stronger adaptation in calcium activity. These results identify activity-dependent patterns of microglial surveillance during ICMS and suggest that stimulation itself can shape microglial contributions to circuit stability and neuroprosthetic performance.

## 2. Methods

### 2.1. Experimental animal models

**Figure 1.**
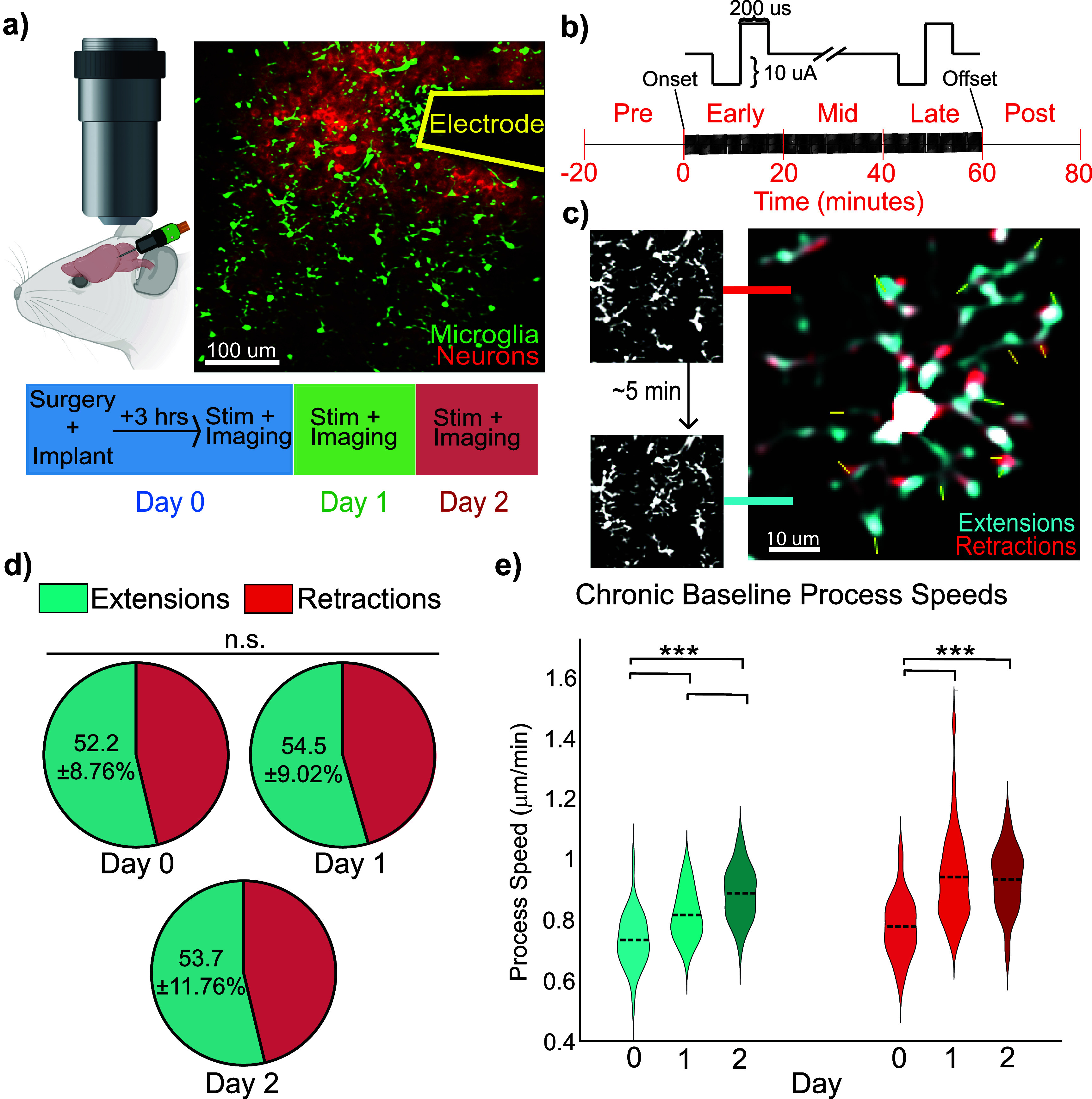
Chronic microelectrode implantation transiently increases microglial process motility across Days 0–2. (a) Experimental setup for two-photon microscopy of Cx3CR1-GFP/Thy1-jRGECO1a mice implanted with a single-shank microelectrode in L2/3 of the visual cortex. (b) ICMS paradigm for 10 Hz symmetric, cathodic-leading, biphasic pulses with a pulse width of 200 *µ*s for 1 h. (c) Representative analysis of microglial process motility using FIJI. Time-lapsed images were overlayed in 5 min intervals to highlight extensions (blue) and retractions (red). (d) The net balance of microglial process movements (extensions vs. retractions) remains unchanged across Days 0–2 post-implantation (*N* = 4 mice; *n* = 39, 38, 37 microglia; raw counts Day 0: 654/583, Day 1: 711/595, Day 2: 747/648); GLMM, *p* = 0.700), reflecting stable overall microglial surveillance. Within-day analyses (supplemental figure 1(b)) reveal a transient bias toward extensions on Day 1. Cumulative extensions and retractions were calculated by summing process movements from four consecutive 5 min intervals for each microglia. (e) Chronic implantation increases extension and retraction rates over days. LME main effect of Day: extensions *p* = 2.04 × 10^−12^, retractions *p* = 4.02 × 10^−09^; post hoc pairwise contrasts: extensions D0vsD1 *p* = 1.9 × 10^−5^, D0vsD2 *p* = 2.6 × 10^−13^, D1vsD2 *p* = 1.9 × 10^−4^; retractions D0vsD1 *p* = 2.2 × 10^−8^, D0vsD2 *p* = 1.0 × 10^−7^, D1vsD2 *p* = 0.78 (*N* = 4 mice).

All animal care and procedures were performed with the approval of the University of Pittsburgh Institutional Animal Care and Use Committee and in accordance with regulations specified by the Division of Laboratory Animal Resources. Mice were kept under standard conditions on a 12 h light/ dark cycle with access to water and food *ad libitum*. Two strains, B6.129P2(Cg)-Cx3cr1^tm1Litt^/J (Cx3cr1^GFP^, strain# 5582, Jackson Laboratories; Bar Harbor, ME) and Tg(Thy1-jRGECO1.1a)GP8.62Dkim/J (jRGECO1a, strain# 30528, Jackson Laboratories; Bar Harbor, ME), were crossed to generate double-transgenic offspring expressing GFP in microglia and jRGECO1a in excitatory neurons (Cx3cr1^GFP^/jRGECO1a, figure 1(a)). This model enabled simultaneous quantification of microglial morphology and neuronal calcium activity, with jRGECO1a providing dense and selective labeling of excitatory neurons in layer 2/3 in V1, consistent with prior characterizations [44].

### 2.2. Probe implantation surgery

Cx3cr1^GFP^/jRGECO1a mice (*n* = 2 male, 2 female, <6 mo., 25–35 g) were implanted with a single-shank Michigan style microelectrode array for awake, head-fixed imaging, as described previously [47–49]. The mice were sedated with a cocktail of 7 mg kg^−1^ and 75 mg kg^−1^ ketamine prior to removal of the sterilized scalp and drilling of bilateral craniotomies over the visual cortices. Bone screws were implanted over the motor cortices for stability and for ground and reference. Prior to implantation, iridium electrode sites were activated to increase charge storage capacity [50] and 1 kHz impedance was verified to be <500 kOhm. Electrodes were targeted to the right hemisphere unless there was bleeding or injury. Single-shank 3 mm long electrode arrays with 4 Iridium channels of 702 *µ*m^2^ electrode sites spaced 50 *µ*m apart (NeuroNexus Technologies, Ann Arbor, MI) were implanted at a 30° angle from the horizontal plane using a Microdrive (MO-81, Narishige, Japan) so that stimulation occurred in layer 2/3 of the visual cortex (final depth of 200–300 *µ*m below the surface). Once the electrode was inserted, the craniotomies were filled with sealant (Kwik-Sil) before being sealed with glass coverslips and dental cement. Ketofen (5 mg kg^−1^) was provided post-operatively up to two days post-surgery or as needed. Animals were allowed to wake up fully prior to any stimulation and imaging (3+ h).

### 2.3. Stimulation paradigm

Stimulation was provided using a TDT IZ2 stimulator controlled by an RZ5D system (Tucker-Davis Technologies, Alachua, FL). The stimulation paradigm is illustrated in figure 1(b) and is biphasic, symmetric, and cathodic-leading with 200 *µ*s pulse width and 10 *µ*A amplitude resulting in 2 nC/phase charge injection, well below the 4 nC/phase safety limit to ensure maximal biocompatibility [51, 52] even for a stimulation trial lasting an hour. Experiments included a 20 min non-stimulation baseline, 60 min of stimulation, and 20 min post-stimulation rest (figure 1(b)).

### 2.4. Two-photon imaging and stimulation

Imaging of neurons and microglia were achieved with a two-photon microscope (Bruker, Madison, WI) with an OPO laser (Insight DS+, Spectra Physics, Menlo Park, CA) equipped with a 16 × 0.8 NA water immersion objective (Nikon Instruments, Melville, NY) with a 3 mm working distance resulting FOV of 407 × 407 *µ*m^2^ (1024 × 1024 pixels). Image acquisition consisted of ZT-series with a 2 *µ*m step size over a 20 *µ*m stack size with a 4.6 *µ*s dwell time resulting in a stack period of 60.4517s. The wavelength of the latter alternated between stacks, starting with 1060 nm to image neuron activity, and 920 nm to image microglia morphology. While this imaging approach reduced temporal resolution, it minimized microglial process displacement across planes, maintained high signal-to-noise ratios for both cell types, and limited thermal loading near the electrode. To assess sampling consistency, we qualitatively confirmed the density of jRGECO1a-positive neurons per imaging volume was consistent across animals and sessions. Furthermore, microglial processes within the imaging volume encountered multiple neurons exhibiting distinct adaptation profiles, enabling an unbiased assessment of interaction frequency across neuronal response types. To minimize edge-related sampling artifacts, microglia located at the boundaries of the imaging volume were excluded from analysis. Imaging time points were 3 h, 24 h, and 48 h post-implantation to assess acute and early chronic responses. Timing metadata for each stack was exported for later synchronization with other datasets.

### 2.5. Data analysis

#### 2.5.1. Microglia analysis

The microglia ZT-series was processed using a Richardson–Lucy total variation deconvolution in ImageJ [53] followed by 3D Gaussian blurring and background subtraction. Following rigid motion correction, the series was then condensed to a 2D plane by taking the average of each Z-stack to capture as many microglia processes as possible regardless of their direction.

Process motility was manually measured in FIJI [54] via overlayed scans taken 4.32 min (2 frames) apart, like previous studies [55], where cyan represents extensions of processes and red represents retractions (figure 1(b)). Under 600%–1200% digital zoom, process migration toward the probe and cell body displacement toward the nearest probe surface were measured using the ‘Measure’ function in ImageJ. To avoid quantifying process movements out of the imaging volume, microglia were included only if they exhibited typical indicators of surveillance [15, 56], including repeated extensions and retractions. Measurements of process movements were verified by viewing the original T-series. Only process movements were captured during these measurements and were manually classified as extensions or retractions. Microglia were excluded from statistical analysis if they exhibited fewer than four measurable process movements during a given imaging session. These exclusions were rare and mostly occurred when cells were partially outside the imaging volume due to *z*-axis truncation. The three cells identified in supplemental figure 2(e) are the only ones excluded by this metric. This threshold was set to ensure robust quantification of process dynamics, as cells with extremely sparse movements due to this volumetric imaging artifact do not provide sufficient data to reliably assess extension/retraction balance, directionality, or polarity indices.

**Figure 2.**
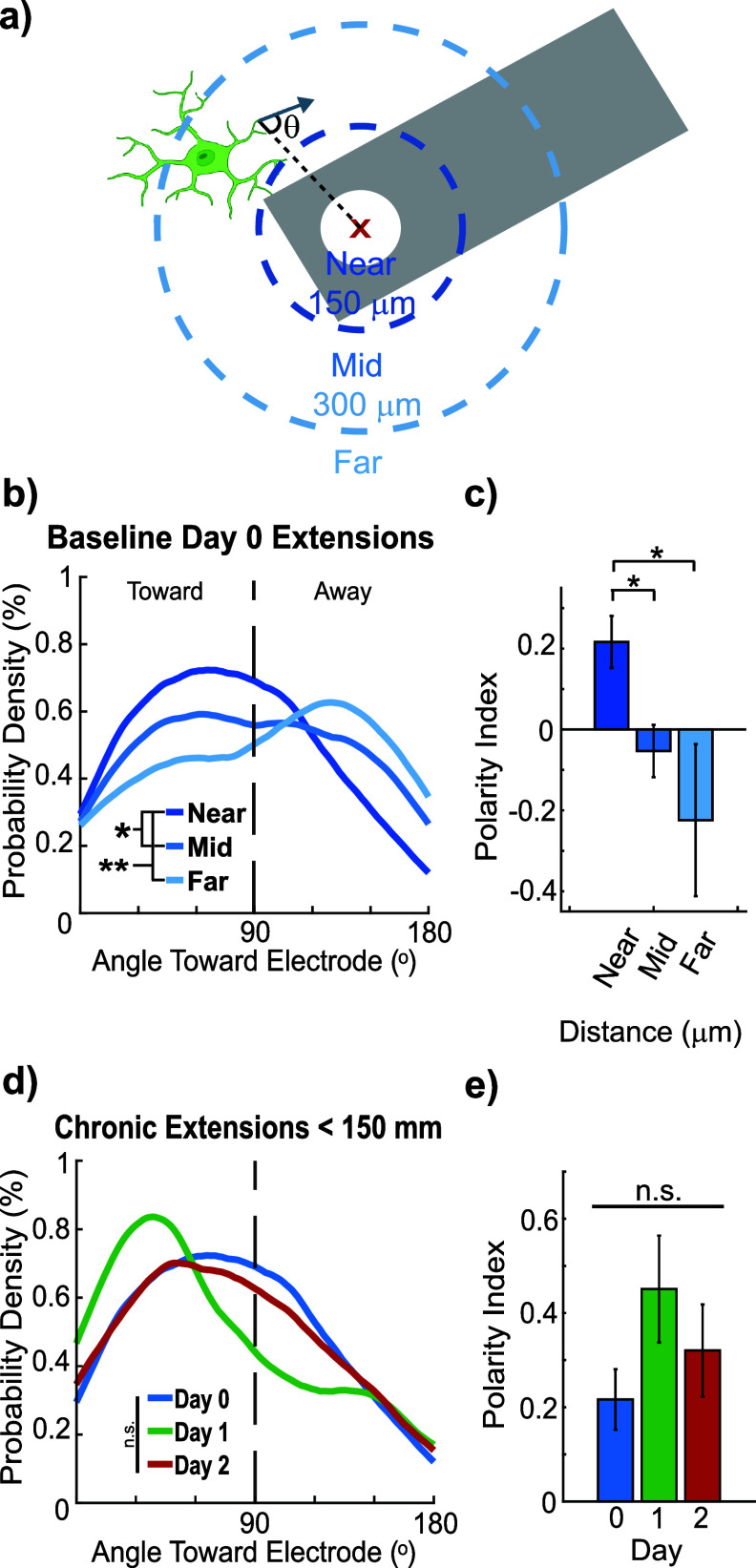
Microglial processes near electrodes transiently orient toward the implant on Day 1. (a) Schematic illustrating how the directionality of microglia process extensions was measured relative to the stimulation site (white circle) of the probe (gray). (b) Distribution of microglia extensions toward the electrode decreases with distance from the electrode (LME main distance effect *p* = 0.0043, post hoc Near: <150 *µ*m, Mid: 150–300 *µ*m, Far: >300 *µ*m; Kolmogorov–Smirnov, *p* = 0.043, 0.0020, 0.29). (c) Microglial process polarity index shows stronger polarization toward the electrode in the <150 *µ*m group compared to mid and far distances (LME main distance effect: *p* = 0.0073. One-way repeated measures ANOVA, *p* = 0.031, 0.021, 0.78; *n* = 18, 27, 6 microglia; microglia with fewer than 4 movements were excluded). (d) For microglia within 150 *µ*m no significant LME main effect of Day was detected (LME Day *p* = 0.15). Distributional comparisons identified a Day 1 vs Day 0 difference (KS *p* = 0.034) but pairwise Day contrasts from the LME were not significant; therefore the Day 1 distributional shift is not supported as a significant Day effect in the mixed-model framework (*n* = 18, 10, 11 microglia; microglia with fewer than 4 movements excluded from polarity index calculations). Microglia with fewer than four measurable process movements or those partially outside the imaging volume were excluded from polarity and directionality analyses to avoid artifacts and ensure reliable quantification of extension behavior.

The properties of these process movements were calculated using MATLAB, and the angle or directionality of the process movements were calculated relative to either the electrode site or the nearest neuronal soma to the process. Each stimulation site was identified manually, and the direction of a process movement was calculated relative to the center of the site. Microglial extensions were analyzed in three distance bins (<150 *µ*m, 150–300 *µ*m, >300 *µ*m) to capture spatially graded responses to electrode implantation. These bins correspond to clusters of microglia with preferential extension toward the probe, partial directional bias, or largely ramified morphology, consistent with prior characterizations of microglial responses to chronic implants [26].

**Figure 3.**
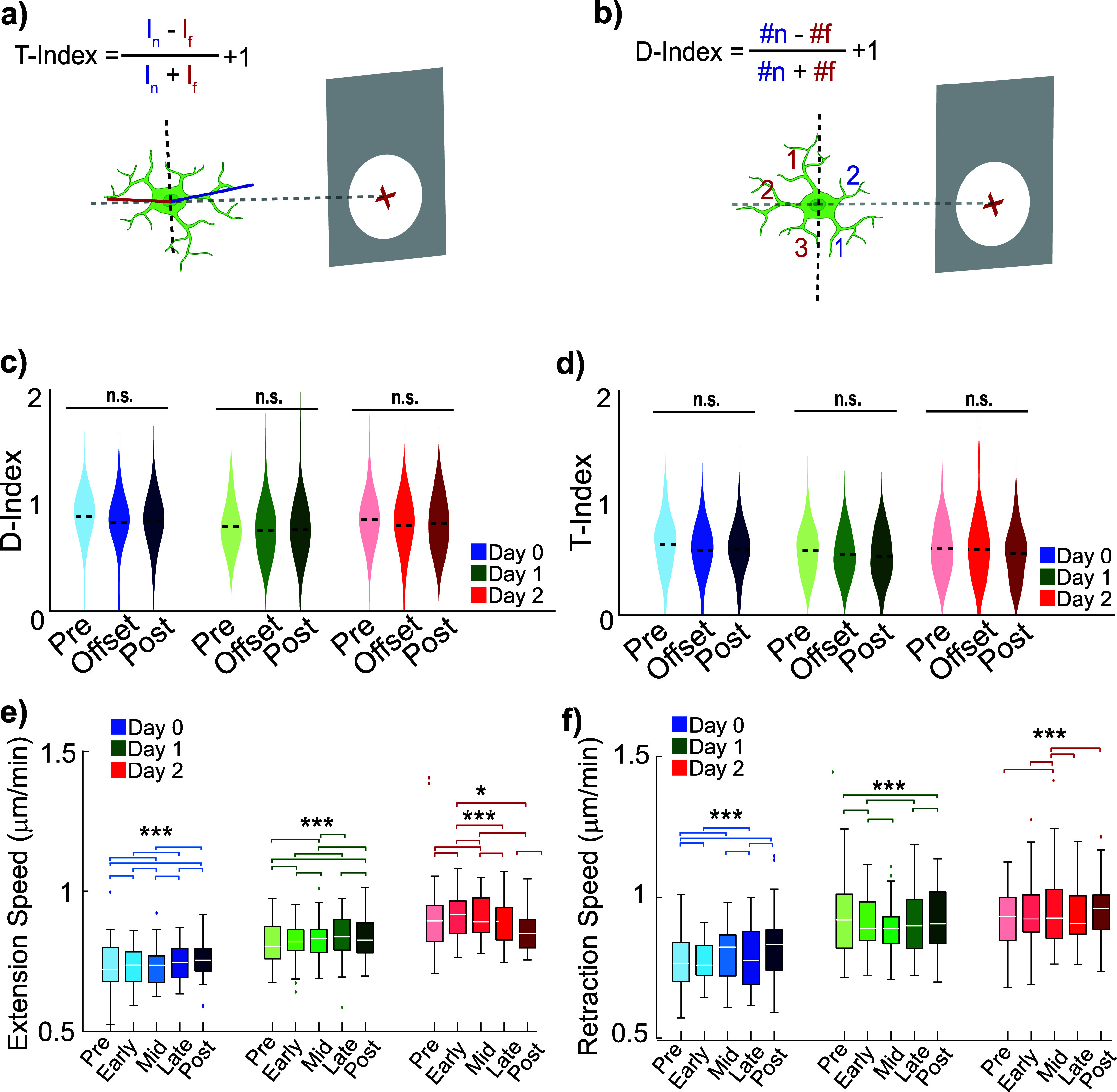
Ten Hz ICMS transiently modulates microglial process speeds without altering overall morphology. (a) *T*-indices represent microglia *T*-stage morphology activation quantified using the length of the processes as a function of distance from the probe (gray). (b) *D*-indices represent microglia directionality of activation quantified using the direction of the processes as a function of distance from the probe (gray). (c) *D*-index not significantly affected by Day, Time, or Day × Time interaction (LME, *p* = 0.81, 0.33, 0.93). (d) *T*-index not significantly affected by Day, Time, or interaction (LME, *p* = 0.65, 0.40, 0.90). (e) Extension speeds show significant Day, Phase, and Day × Phase effects (LME: Day *p* = 4.1 × 10^−15^; Phase *p* = 3.0 × 10^−26^; Day × Phase *p* = 1.4 × 10^−55^); post hoc contrasts shown. (f) Retraction speeds also show significant Day, Phase, and Day × Phase effects (LME: Day *p* = 4.5 × 10^−12^; Phase *p* = 2.0 × 10^−17^; Day × Phase *p* = 2.2 × 10^−33^). (e), (f) post hoc paired comparisons within each day were conducted using LME with Holm correction for multiple testing. *= *p* < 0.05, **= *p* < 0.01, ***= *p* < 0.005.

Similarly, process movements relative to the nearest neuron were calculated according to the center of the soma. Lastly, Directionality- and Transitional-indices (*D*- and *T*- indices) were calculated for each cell (equations (1) and (2)) [49, 57–59] separately with the length and position of processes manually drawn in ImageJ and processed in MATLAB (figure 3(b)),

\begin{equation*}D{ } = { }\frac{{\left( {f - n} \right)}}{{\left( {f + n} \right)}} + 1\end{equation*}

where *f* = # of processes away from electrode, *n* = # of processes near electrode,

\begin{equation*}T{ } = { }\frac{{\left( {f - n} \right)}}{{\left( {f + n} \right)}} + 1\end{equation*}

where *f* = length of longest process away from electrode, *n* = length of longest process near electrode.

In order to assess the directionality of process movements for a single microglia cell, a histogram of the angles of the process movements relative to the electrode or nearest neuron was plotted. This histogram was then fit with a probability distribution function using an Epanechnikov kernel function. From there, the area under the curve (AUC) of the probability distribution function was calculated, with the area from 0 to 90 counting as toward the electrode, and 90–180 counting as away from the target. The polarity-index (*ρ*-index) was then calculated as a proportion of movements toward and away from the target (equation (3)) and ranges from −1 to 1, where −1 indicates a microglia only extending processes away from the target and 1 represents a microglia only moving processes toward the target,

\begin{equation*}\rho { } = { }\frac{{\left( {t - a} \right)}}{{\left( {t + a} \right)}}\end{equation*}

where *t* = AUC of movements toward target, *a* = AUC of movements away from target.

#### 2.5.2. Neural activity analysis

For each stimulation session, the average of each motion-corrected 1060 nm Z-stack was used to manually identify neurons and their profiles outlined using the ROI manager in ImageJ [10]. These ROIs and full ZT-series were subsequently imported into MATLAB for further fluorescence analysis. The fluorescence intensity of the neurons was filtered by taking the maximum intensity in each Z-stack to ensure that signals from somas not included in the full 20 *µ*m stack would not be adversely represented. The resulting fluorescent signal (*F*) had a sample for every 2.16 min representing the maximal neuronal calcium activity. To correct for noticeable photobleaching due to the length of the imaging session (100 min), the fluorescent signal for the full FOV of each imaging session was fit with a linear model excluding any stimulation timepoints. Each neuron’s fluorescent signal was then adjusted according to the linear model. Beyond the signal preprocessing, each neuron’s distance from the stimulation site was calculated for further spatiotemporal analysis.

Once the neuron signal has been preprocessed, the neuron was classified according to its activation and the subsequent temporal profile. Given that microglia participate in neuronal excitability through fractalkine and purinergic signaling [24, 27, 40, 46], identifying the behavior of a neuron provides more specific insight into how microglia may modulate its excitability. A neuron was considered activated if its change in fluorescence (Δ*F*/*F*_0_) exceeded three standard deviations above the mean fluorescence during the baseline period, where *F*_0_ is the mean baseline fluorescence over the 20 min pre-stimulation period. This thresholding approach has been widely used in calcium imaging analyses to ensure that only responses exceeding intrinsic baseline variability are classified as activation [8, 10]. ΔF/F_0_ was calculated for each ROI relative to this baseline to quantify changes in calcium activity during ICMS. Subsequent profile analyses determined 4 distinct profiles of activated neurons: depressed, baseline adapting, adapting, and non-adapting. Neurons were identified as depressed post-activation if the average calcium activity was less than −0.5 times the threshold identified earlier. Neurons were classified as baseline adapting if the average of the signal during stimulation was within one standard deviation of the baseline. Neurons were adapting if the average of the signal was between 1 and 3 standard deviations of the activation threshold. Finally, non-adapting neurons were identified if more than half of the stimulation timepoints exceeded the activation threshold, suggesting these neurons were active for more than 30 min. These classification criteria were mutually exclusive. Sample waveforms for these activation profiles are shown in figure 6(b).

Non-activated neurons also exhibited distinct temporal profiles, with a subset showing immediate depressed activity at the onset of stimulation. These depressed non-activated neurons (supplemental figure 6(b)) were defined as having average calcium activity less than −0.5 times the activation threshold, without ever exceeding the activation criterion. This contrasts with depressed post-activation neurons, which first crossed the activation threshold before subsequently falling below baseline activity.

While neurons exhibited distinct activation profiles during stimulation, there were also two distinct profiles of neuronal calcium activity post-stimulation (figure 8(a)). Neurons were classified as depressed post-stimulation if the average calcium activity was less than −0.5 times the activation threshold. Otherwise, neurons were classified as baseline post-stimulation when the activity was within 0.5 standard deviations of the baseline activity.

#### 2.5.3. Microglia–neuron interaction analysis

To examine microglia–neuron interactions during stimulation, microglia were overlaid with the neuronal ROIs identified earlier and each interaction was manually recorded alongside the timing of the microglia process movement. ROIs were designed to include regions with a representative density of jrGeco1a-expressing neurons and evaluated microglial targeting behavior only when clear spatial proximity to known neurons existed. Interactions were classified into connections and disconnections whenever microglia processes touched a neuron soma or retracted away from the soma. It is crucial to note that any disconnections would require the microglia to already be in contact with the neuronal soma.

#### 2.5.4. Statistics

All statistical analyses were performed in MATLAB with an alpha level of 0.05, and Bonferroni or Holm corrections were applied for multiple comparisons where appropriate. Homogeneity of variance was formally tested using Levene’s Absolute Test, and normality of residuals was assessed for all parametric analyses. When assumptions of normality or homogeneity of variance were violated, non-parametric alternatives were applied. Specifically, one-way Kruskal–Wallis tests replaced ANOVA for continuous data, and Kolmogorov–Smirnov tests were used for distributional and angular measures. Microglial dynamics within each post-implantation day were analyzed using repeated-measures ANOVA or equivalent non-parametric tests to account for multiple measurements from the same microglia. All post hoc comparisons were conducted with Bonferroni or Holm correction within the repeated-measures framework to control for multiple testing. These repeated-measures analyses explicitly account for correlation between observations from the same microglia across time and do not assume statistical independence of repeated measurements. Day was included as a fixed factor (in addition to angle, trial phase, distance, and/or activity as appropriate) in all models to account for temporal structure and injury-related variance, preserving the correlation structure across days and ensuring that within-day effects are not treated as statistically independent. However, day effects were treated as contextual variables and not interpreted as primary outcomes, as the study was designed to evaluate stimulation-dependent modulation of microglial behavior within distinct post-implantation states. Longitudinal effects across days were assessed using linear mixed-effects (LMEs) models for continuous measures, with microglia identity treated as a random effect to account for intra-subject correlation. When data were unbalanced or represented counts or proportions, generalized LMEs models (GLMMs) with a binomial distribution and logit link were applied. The choice of LME versus GLMM was determined by data type and residual distribution. These LME-based results supersede one-way ANOVA and Kruskal–Wallis tests, providing a framework that properly accounts for repeated measures across microglia and days. Violin plots ([60] Violin plots for Matlab) depict the mean as a horizontal line and error bars represent standard error unless otherwise specified. All non-parametric and mixed-effects analyses include multiple comparison correction and are explicitly linked to the corresponding figures and results.

## 3. Results

Two-photon imaging in Cx3cr1^GFP^/jRGECO1a mice enabled simultaneous tracking of neuronal calcium activity and microglial dynamics during prolonged 10 Hz ICMS (1 h). Microglial processes traversed the full imaging volume and encountered neurons with diverse adaptation profiles, enabling unbiased interaction analysis. Microglia at volume edges were excluded to avoid sampling artifacts. Four distinct neuronal adaptation profiles were identified: (a) *non-adapting* neurons maintaining suprathreshold activity throughout 60 min stimulation, (b) *adapting* neurons showing progressive activity reduction but remaining above baseline, (c) *baseline-adapting* neurons returning to pre-stimulation activity levels during stimulation, and (d) *depressed* neurons exhibiting sub-baseline activity post-activation. Microglial processes dynamically responded to ICMS, extending toward stimulation sites and active neurons, with microglia–neuron contact frequency inversely correlating with neuronal adaptation magnitude. Mechanistic insights were further gained by comparing ICMS during the acute (3 h) and chronic (24–48 h) post-implantation responses, which revealed that microglial encapsulation of electrodes coincided with neuronal hypoactivity. Using cellular-resolution imaging and biphasic stimulation (2 nC/phase; within established safety thresholds [61]), we demonstrate that microglia may modulate ICMS-induced network activity. These findings challenge neuron-centric paradigms and support a model in which glia function as computational elements influencing stimulation efficacy.

### 3.1. Chronic microelectrode insertion drives and directs faster microglial process movements

Prior studies have shown that microglia extend processes toward newly implanted electrodes within the first few hours [6, 26]. To isolate the contribution of microglia to the neuronal response during ICMS, it was necessary to first characterize microglial behavior following chronic electrode implantation in the absence of stimulation (figure 1). All measurements in this section reflect pre-stimulation baselines, ensuring that observed behaviors represent chronic implantation effects alone and providing a reference for evaluating microglia–neuron interactions specific to ICMS.

When assessed cumulatively across days, microglial surveillance behavior, quantified as the extension-to-retraction ratio, remained stable (figure 1(d); GLMM, *p*= 0.700), indicating preservation of the overall balance between opposing process movements despite electrode implantation. In contrast, the total number of process movements per microglia exhibited a significant effect of Day (supplemental figure 1(a); LME marginal ANOVA, *F* = 9.8, *p*= 1.2 × 10^−4^), reflecting a progressive increase in overall motility following implantation. All within-day analyses were conducted using repeated-measures models that account for correlations between measurements from the same microglia and within the same animal, ensuring valid inference despite repeated sampling across days. Post hoc comparisons revealed a significant increase in total movements by Day 2 relative to Day 0 and Day 1 (GLMM pairwise tests: D0 vs D2 *p* = 2.5 × 10^−5^, D1 vs D2 *p* = 0.015), while the difference between Days 0 and 1 did not reach significance (*p* = 0.054). Repeated-measures corrections were applied to account for multiple process events originating from the same microglia. This day-specific deviation is consistent with an acute response to electrode insertion, potentially reflecting the combined influence of residual anesthesia and early inflammatory signaling associated with implantation [62–64]. Importantly, despite increased overall motility by Day 2, the extension-to-retraction ratio remained independent of microglial distance from the electrode (supplemental figure 1(d)), indicating that changes in movement frequency reflect a global injury-related response rather than spatially biased surveillance [31, 65]. Together, these analyses demonstrate that electrode implantation induces a time-dependent increase in microglial process activity without disrupting the long-term balance between extensions and retractions in surveilling microglia, with transient, day-specific deviations reflecting acute injury responses rather than sustained directional bias.

Instead, microglia behavior following electrode implantation is primarily expressed through changes in process movement speed. Using LMEs models that account for repeated measures within microglia, we found a significant main effect of Day on overall process speed (LME: *F* = 34.6, *p*= 2.0 × 10^−12^ for extensions; *F* = 23.1, *p*= 4.0 × 10^−9^ for retractions), as well as a main effect of Type (extensions vs. retractions; *F* = 4.2, *p*= 0.040) and a Day × Type interaction (*F* = 4.3, *p*= 0.014), indicating that day-dependent modulation differs between extension and retraction velocities. Consistent with these results, mean extension speeds increased from Day 0 to Day 2 (Day 0: 0.73 ± 0.02 *µ*m min^−1^, Day 1: 0.82 ± 0.02 *µ*m min^−1^, Day 2: 0.89 ± 0.022 *µ*m min^−1^), whereas retraction speeds showed a more rapid increase followed by a plateau (Day 0: 0.78 ± 0.022 *µ*m min^−1^, Day 1: 0.94 ± 0.03 *µ*m/min, Day 2: 0.93 ± 0.03 *µ*m min^−1^) (figure 1(e)). Across all days, retractions occurred at slightly higher velocities than extensions, despite extensions being more frequent than retractions (supplemental figure 1(c)). In contrast to the strong temporal dependence, neither extension nor retraction velocities were significantly associated with distance from the electrode insertion site (supplemental figure 1(e)), suggesting that speed changes reflect a global injury-related response rather than spatially graded targeting.

Having characterized the overall dynamics of microglial process movements post-implantation, we next examined the spatial orientation of these processes relative to the electrode to capture directional responses to injury (figure 2(a)). Microglia were grouped into three distance ranges (<150 *µ*m, 150–300 *µ*m, >300 *µ*m) to capture clusters with preferential extension toward the implant, partial directional bias, or largely ramified morphology, consistent with prior characterizations of microglial responses to chronic implants [26]. Distinct behavioral changes were observed based on the distance of microglia from the electrode and directionality of process extensions relative to the electrode were quantified.

Microglia located within 150 *µ*m of the electrode extended more frequently toward the electrode than microglia at greater distances (figure 2(b)). There was a significant main effect of Distance on extension distribution (LME main effect Distance *p* = 0.0043). Kolmogorov–Smirnov tests for distributional differences by distance yielded Near vs Mid *p* = 0.043, Near vs Far *p* = 0.0020, Mid vs Far *p* = 0.29. The polarity index also showed a significant main effect of Distance (LME main effect *p* = 0.0073) with stronger polarization in the <150 *µ*m group. For directional analyses, we focused on extension events because retractions were generally oriented opposite to extensions and therefore largely mirrored extension orientation, contributing little independent information about directional bias (supplemental figures 2(a) and (b)). Directional targeting was quantified using a polarity index computed from extension angles (positive = toward electrode; negative = away; equation (3), supplemental figures 2(c) and (d)).

To formally assess directional bias while accounting for repeated measurements from individual microglia, we fitted a LMEs model with Distance as a fixed effect and MicrogliaID as a random effect. This analysis revealed a significant effect of distance on polarity (*F* = 5.5, *p*= 0.0073), with microglia in the Mid (150–300 *µ*m) and Far (>300 *µ*m) bins exhibiting significantly lower polarity than those in the Near (<150 *µ*m) bin (figure 2(c); polarity index mean ± SEM: Near 0.216 ± 0.0643; Mid −0.053 ± 0.0647; Far −0.224 ± 0.1879). These results confirm that extensions of microglia within 150 *µ*m are preferentially directed toward the electrode and that this directional bias is robust to repeated measures. Because these biases were observed at baseline, they likely reflect the local injury caused by electrode insertion rather than stimulation-driven targeting.

In contrast, for microglia within 150 *µ*m of the electrode, a distributional shift in extension angles was detected on Day 1 relative to Day 0 (Kolmogorov–Smirnov test, *p* = 0.034), indicating a transient bias in process orientation. However, a LMEs model accounting for repeated measurements within microglia and animals did not detect a significant main effect of Day on the polarity index (LME, *p* = 0.15). Thus, while directional shifts are evident at the distributional level, they are not robust when controlling for within-cell and within-animal dependence. Taken together, these results indicate that the spatial bias of extensions toward the electrode is a robust feature of the local injury response, whereas temporal dynamics of extension orientation are minimal.

Interestingly, the number of extensions toward the electrode for microglia within 150 *µ*m peaked on Day 1 (figure 2(e), *n* = 18, 10, and 11 microglia; polarity index: Day 0: 0.22 ± 0.06, Day 1: 0.45 ± 0.11, Day 2: 0.32 ± 0.10). These results suggest that the distribution of microglia extensions toward the electrode is influenced by the injury response, though additional factors such as surrounding tissue and cellular behavior may also modulate microglial process orientation.

### 3.2. Prolonged low frequency ICMS does not affect microglia morphology, but modulates and directs process extensions

Previous studies suggested that low-frequency electrical stimulation can attenuate microglial activation post-stimulation, potentially mitigating inflammatory consequences [14, 66, 67]. However, whether stimulation alters microglial inflammatory state during ongoing ICMS, particularly in the context of electrode-induced injury, remains unresolved. Microglial morphology provides a direct readout of activation state, with homeostatic microglia exhibiting highly ramified processes and activated microglia adopting a less branched, amoeboid morphology [15, 26, 68]. To assess whether prolonged low-frequency ICMS alters microglial morphology during stimulation, we quantified directionality- and transitional-indices (*D*- and *T*-indices) [59] (figure 3(b)), which capture process orientation and length dynamics as a function of distance from the electrode.

Because microglial behavior evolves rapidly following electrode implantation due to injury-induced inflammation and tissue remodeling [57], post-implantation day was treated as a biologically meaningful contextual variable rather than a nuisance factor. Critically, our primary hypothesis tested whether ICMS at a lower phase modulates microglial morphology, whereas distance- and day-specific analyses were performed to contextualize implantation-associated spatial heterogeneity. LMEs models incorporating Day, Time, and their interaction revealed no significant effects of ICMS on microglial process directionality or length across the population (figures 3(c) and (d)). Specifically, *D*-index showed no significant main effects of Day or Time, nor a Day × Time interaction (LME: Day *p*= 0.81, Time *p* = 0.33, Day × Time *p* = 0.93), indicating that 1 h of 10 Hz ICMS does not produce sustained changes in microglial process orientation. Similarly, *T*-index exhibited no significant effects of Day, Time, or their interaction (*T*-index LME: Day *p* = 0.65, Time *p* = 0.40, Day × Time *p* = 0.90), demonstrating that ICMS does not significantly modulate microglial process length relative to the electrode.

In contrast, supplemental analyses addressed distinct questions related to spatial and temporal heterogeneity following implantation rather than the presence of stimulation. Supplemental figures 3(a) and (c) examined whether *D*- and *T*-indices vary across post-implantation days independent of stimulation, whereas supplemental figures 3(b) and (d) test for distance-dependent effects within individual days. Within these focused analyses, transient and spatially restricted effects were observed. On Day 1, microglia located closer to the electrode exhibited lower *D*- and *T*-indices (supplemental figures 3(b) and (d); *D*-Index: *p* = 0.046; *T*-Index: 150–300: *p* = 0.0034, >300: *p* = 0.0007). This pattern is consistent with prior studies indicating that microglia near implanted electrodes undergo morphological changes in response to insertion-related tissue damage [26]. A transient decrease in T-index was also observed on Day 1 between stimulation onset and offset for microglia located >300 *µ*m from the electrode (supplemental figure 3(f); *p* = 0.0084). These effects were not sustained across days and did not emerge as significant ICMS effects when analyzed using mixed-effects models that account for repeated measures across cells and animals.

Day 0 measurements may additionally be influenced by residual anesthesia, which can transiently suppress microglial motility and obscure early post-implantation effects. Taken together, these analyses demonstrate that while implantation induces transient, distance-dependent morphological heterogeneity, prolonged low-frequency ICMS does not drive consistent or population-level changes in the morphology of surveilling microglia. This interpretation aligns with prior observations that evoked and spontaneous neural activity near implanted electrodes is initially elevated post-implantation but diminishes over subsequent days due to inflammatory processes, with only partial recovery [31, 65].

Since ICMS did not produce consistent or sustained changes in the static measure of microglial morphology, we further examined whether stimulation modulated microglial process dynamics. Here, *Phase* is defined relative to stimulation timing, with Pre corresponding to the pre-stimulation baseline, Early, Mid, and Late corresponding to consecutive 20 min segments during the 1 h 10 Hz ICMS period, and Post corresponding to the period immediately following stimulation offset. LMEs modeling revealed that both extension and retraction speeds were significantly modulated in a manner dependent on post-implantation day and stimulation phase, despite the absence of accompanying changes in morphology-based activation indices.

For process extensions, the LMEs model with identified significant main effects of Day (*p* = 4.1 × 10^−15^) and Phase (*p* = 3.0 × 10^−26^), as well as a strong Day × Phase interaction (*p* = 1.4 × 10^−55^), indicating that stimulation-related changes in extension speed were contingent on the evolving post-implantation inflammatory environment. Post hoc paired comparisons were conducted with Holm correction to control for multiple testing, demonstrating robust phase-specific modulation across all days, with specific phase differences within each day remaining significant after adjustment. On Day 0, extension speeds differed significantly between Pre vs Early (*p* = 2.6 × 10^−04^), Pre vs Mid (*p* = 8.9 × 10^−16^), Pre vs Post (*p* = 5.5 × 10^−06^), Early vs Mid (*p* = 8.2 × 10^−06^), Early vs Late (*p* = 7.9 × 10^−04^), Mid vs Late (*p* = 1.1 × 10^−14^), Mid vs Post (*p* = 3.2 × 10^−04^), and Late vs Post (*p* = 2.7 × 10^−05^). A similar pattern was observed on Day 1, with significant differences for Pre vs Early (*p* = 1.8 × 10^−15^), Pre vs Mid (*p* = 3.31 × 10^−03^), Pre vs Post (*p* = 1.3 × 10^−11^), Early vs Mid (*p* = 2.2 × 10^−07^), Early vs Late (*p* = 1.6 × 10^−16^), Mid vs Late (*p* = 1.1 × 10^−03^), Mid vs Post (*p* = 1.8 × 10^−04^), and Late vs Post (*p* = 1.5 × 10^−12^). On Day 2, extension speeds were significantly reduced following stimulation offset, with differences observed for Pre vs Early (*p* = 3.4 × 10^−06^), Pre vs Mid (*p* = 3.6 × 10^−05^), Early vs Mid (*p* = 9.6 × 10^−18^), Early vs Late (*p* = 2.3 × 10^−04^), Early vs Post (*p* = 2.3 × 10^−02^), Mid vs Late (*p* = 5.9 × 10^−08^), and Mid vs Post (*p* = 3.2 × 10^−10^). Collectively, these results indicate that ICMS may be associated with changes in the dynamics of microglia process extensions. The most pronounced and consistent reductions emerging on Day 2 suggest that, when acute injury-related responses have partially stabilized, ICMS offset may suppress microglia extensions.

Retraction dynamics exhibited a complementary but temporally distinct pattern. LMEs modeling revealed significant main effects of Day (*p* = 4.5 × 10^−12^) and Phase (*p* = 2.0 × 10^−17^), as well as a significant Day × Phase interaction (*p* = 2.2 × 10^−33^), indicating that retraction speed modulation was similarly dependent on both stimulation timing and post-implantation state. On Day 0, retraction speeds differed significantly between Pre vs Early (*p* = 5.9 × 10^−08^), Pre vs Mid (*p* = 3.7 × 10^−11^), Pre vs Post (*p* = 4.3 × 10^−09^), Early vs Late (*p* = 1.1 × 10^−06^), Mid vs Late (*p* = 1.3 × 10^−09^), and Late vs Post (*p* = 9.5 × 10^−08^), all confirmed with Holm correction to control for multiple comparisons, consistent with heightened microglial motility during the acute post-implantation phase. On Day 1, significant differences persisted for Pre vs Early (*p* = 1.1 × 10^−07^), Pre vs Post (*p* = 2.1 × 10^−03^), Early vs Mid (*p* = 7.5 × 10^−05^), Early vs Late (*p* = 9.7 × 10^−10^), and Late vs Post (*p* = 6.9 × 10^−05^). By Day 2, retraction-related effects were more restricted, with significant differences observed for Pre vs Mid (*p* = 2.9 × 10^−06^), Early vs Mid (*p* = 3.4 × 10^−07^), Mid vs Late (*p* = 7.1 × 10^−05^), and Mid vs Post (*p* = 4.71 × 10^−08^). Importantly, these robust, phase-specific changes in process motility occurred in the absence of sustained changes in microglial morphology, as quantified by *D*- and *T*-indices. Together with the transient, distance-dependent effects during early post-implantation periods (supplemental figure 3), these findings support a model in which low-frequency ICMS interacts with the evolving inflammatory milieu to bias microglial process dynamics, rather than driving persistent morphological activation. This dissociation between morphology and motility suggests that microglia remain structurally homeostatic while dynamically responsive to stimulation offset and post-implantation state. As such, further investigation into the effects of ICMS on the dynamic responses of surrounding microglia were conducted with environment features used to contextualize the results.

**Figure 4.**
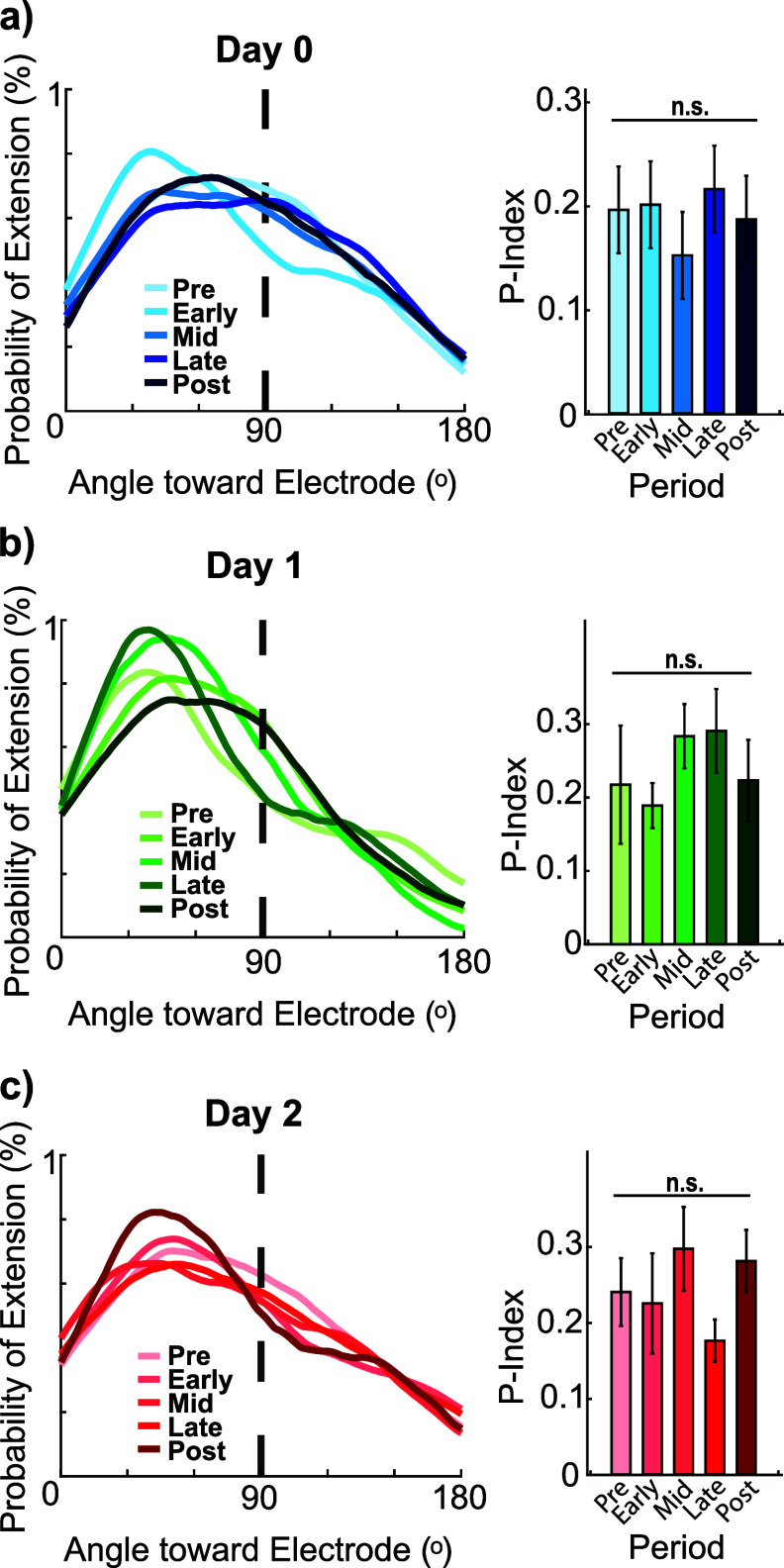
Microglial process orientation toward the electrode does not differ across days during 10 Hz ICMS. (a) On Day 0, the onset of ICMS drives increased microglia processes within 150 *μ*m of the electrode to extend more frequently toward the electrode (Kolmogorov–Smirnov, *p* = 0.026), but does not significantly increase polarization (repeated measures ANOVA, *p* = 0.86). (*N* = 39 microglia, *n* = 194, 174, 187, 187, 165 process extensions). (b) By Day 1, microglia process extensions within 150 *µ*m are not significantly driven toward the electrode, nor polarized by stimulation (*N* = 38 microglia, *n* = 106, 108, 96, 96, 73 process extensions, Kolmogorov–Smirnov, *p* = 0.32 and repeated measures ANOVA, *p* = 0.23). (c) Similarly, on Day 2, ICMS does not significantly drive microglia extensions toward the electrode, nor affect polarization (*N* = 37 microglia, *n* = 132, 71, 88, 99, 93 process extensions, Kolmogorov–Smirnov, *p* > 0.21 and repeated measures ANOVA, *p* = 0.29).

Given that stimulation demonstrated some effects on microglia process behavior, we further investigated whether these behavioral changes might be related to microglia extensions in relation to the site of stimulation. On Day 0, stimulation onset transiently increased extensions toward the electrode without significantly altering polarity (figure 4(a)). For both Day 1 and 2, microglial extensions were already predominantly directed toward the electrode site, and ICMS did not further shift their distribution or polarity (figures 4(b) and (c)). Consistent with figure 2, stimulation-related changes in microglial orientation were not significant at the population level when accounting for repeated measures, despite transient distributional shifts observed on Day 1. Retractions largely opposed extensions and exhibited no directional changes (supplemental figures 4(a)–(c)). These findings suggest that microglia respond to the combination of injury and electrical stimulation, but ICMS alone does not consistently drive process targeting toward the electrode. Neuron-mediated signaling, such as fractalkine release, may also contribute to the observed patterns given the higher neuronal activity near the electrode.

### 3.3. Microglia process extensions migrate toward neuronal activation during low frequency ICMS

**Figure 5.**
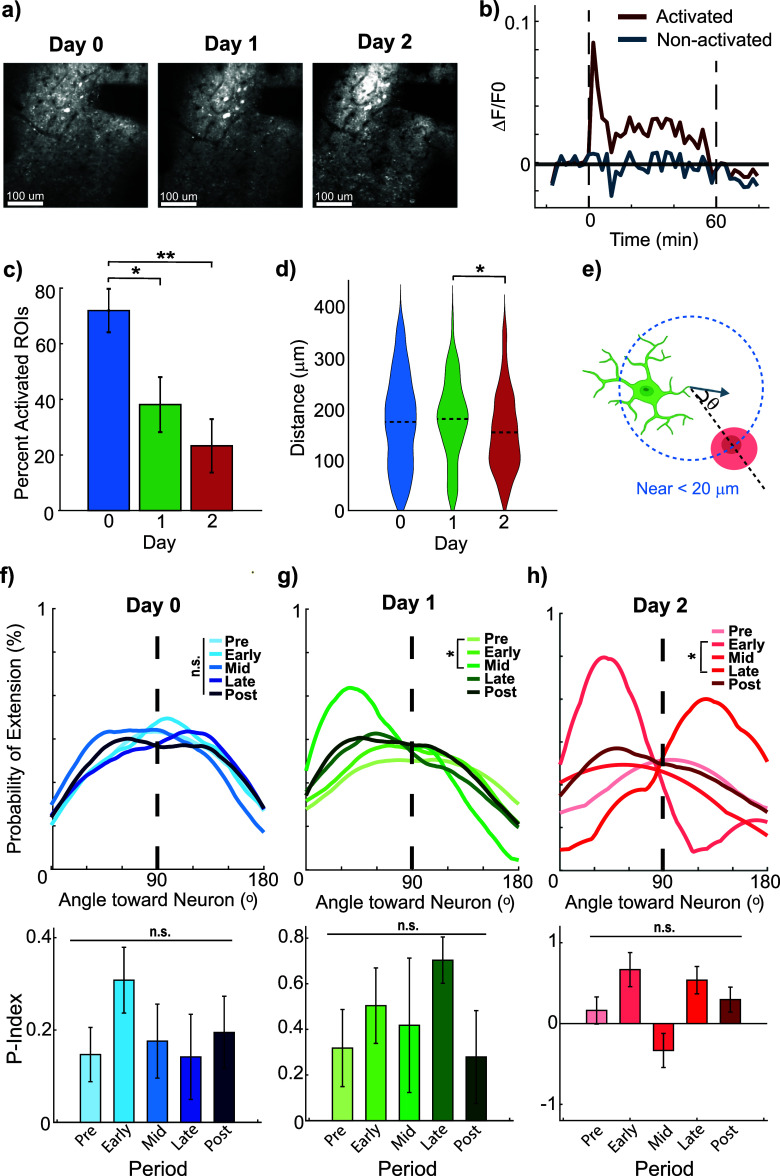
Chronic 10 Hz ICMS reduces neuronal activation and transiently directs microglial processes toward activated neuron. (a) Representative chronic images of activated neurons (Thy1-jRGECO1a). (b) Representative calcium traces of neurons, defined as activated and non-activated. (c) The percentage of activated neurons decreases significantly between Days 0 and 2 (*N* = 4 mice, LME main effect of Days *p* = 0.0053, post hoc *p* = 0.014 (D0vsD1), 0.0019 (D0vsD2), 0.22 (D1vsD2)). (d) Neuron activation shifts closer to the electrode site by Day 2 (*N* = 4 mice, *n* = 335, 140, 83 neurons, One-Way repeated measures ANOVA, *p* = 0.040). (e) Microglia process extensions within 20 *µ*m of the nearest neuron were considered nearby. (f) Day 0: Microglia process extensions are not preferentially directed toward activated neurons (*N* = 39 microglia, *n* = 214, 184, 159, 193, 177 process extensions Kolmogorov–Smirnov, *p* > 0.27, Repeated Measure ANOVA, *p* = 0.23). (g) Day 1: Microglia increased extensions toward activated neurons after 20 min of ICMS (*N* = 38 microglia, *n* = 52,44,48,40,42 process extensions, Kolmogorov–Smirnov, *p* = 0.021) but are not significantly polarized (Repeated Measure ANOVA, *p* = 0.15) (h) Day 2: Microglia show initial extensions toward activated neurons at onset shift away after 40 min (*N* = 37 microglia, *n* = 6, 13, 3, 12, 9 process extensions, Kolmogorov–Smirnov, *p* = 0.002). Overall, during stimulation, microglia are not significantly polarized toward activated neurons (repeated measure ANOVA, *p* = 0.31). Non-activated neurons were excluded from the main plots due to minimal calcium signal; their corresponding microglia interactions are presented in supplemental figure 5.

Given the observed shifts in microglia process behavior in response to ICMS, we next explored how these behaviors are linked to neuronal activity. Fewer neurons were identified as activated over days (LME main effect Days *p*= 0.0053), with a significant decrease (*p* = 0.0019) between Day 0 and 2 post-implantation (figure 5(c), Day 0: 71.9 ± 7.8%, Day 1: 38.1 ± 9.9%, Day 2: 23.3 ± 9.6%). The average distance of activated neurons from the electrode also decreased by Day 2 (figure 5(d), Day 0: 173.4 ± 5.0 *µ*m, Day 1:179.1 ± 6.7 *µ*m, Day 2: 153.0 ± 8.4 *µ*m), consistent with prior observations that the inflammatory environment around an implanted electrode diminishes neuronal responsiveness over the first few days post-implantation [31, 62, 65, 69].

To assess microglia responses to neuronal activity, processes were classified as ‘near’ activated neurons if within 20 *µ*m, thus capable of interacting with the neurons during any of the 20 min periods of stimulation (figure 5(e)), a threshold compatible with their measured maximum process speed of 0.94 ± 0.02 *µ*m min^−1^ (figure 1(d)). Interestingly, on Days 1 and 2, microglia extended processes toward activated neurons significantly more frequently during stimulation (figure 5(g); extension *p*= 0.021, polarity index *ρ* = 0.15; figure 5(h); extension *p*= 0.002, polarity index *ρ* = 0.31). Despite the increased frequency of extensions, polarity indices did not show a statistically significant shift. This likely reflects the averaging of directional vectors across heterogeneous targets (i.e. activated neurons positioned in different orientations and the electrode itself), which dilute neuron-specific biases when considered at the population level. Non-activated neurons were analyzed separately (supplemental figures 5(a)–(c)), which confirmed that ICMS did not significantly drive microglial process extensions, retractions, or polarization near these neurons. Microglia–neuron interactions during ICMS preferentially engage neurons exhibiting activity changes rather than quiescent neurons, highlighting this distinction.

These day-specific observations indicate that microglia responsiveness to neuronal activation emerges after the acute injury phase, rather than being driven directly by ICMS itself. The transient nature of this response aligns with the declining availability of activated neurons over Days 1–2, reinforcing the importance of separating within-day effects from cumulative, multi-day trends in interpreting microglial behavior.

### 3.4. Neuronal activity decreases with increasing microglia–neuron interactions

**Figure 6.**
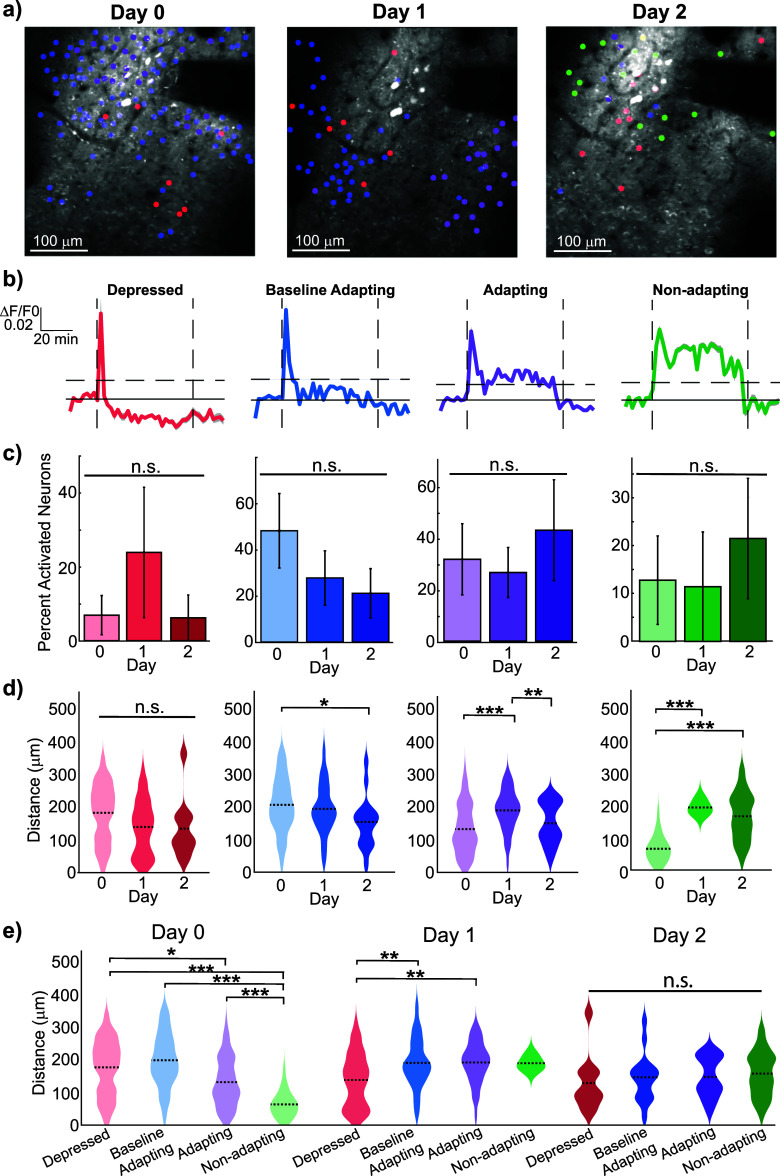
Spatial distribution of neuron activation profiles during sustained 10 Hz ICMS across Days 0–2. (a) Representative chronic images of activated neuron profiles for spatial context. Note that not all neuron types are visible in every frame; some profiles (e.g. non-adapting neurons) may appear in other imaging frames. (b) Representative calcium traces of differentiated neuron profiles, including stimulation onset (1st vertical dashed line), offset (last vertical dashed line), and activation threshold (horizontal dashed line). (c) In contrast, the percentages of identified neuron profiles do not significantly vary across days (*N* = 4 mice, *p* = 0.54, 0.087, 0.74, 0.73). (d) Significant differences in neuronal response distances were observed across days (LME effect Days *p* = 2.7 × 10^−19^). The neuron profiles exhibit less adaptation during the 1 h ICMS period are located further from the electrode across 2 d (*N* = 4 mice, Depressed *n* = 29, 39, 10, Baseline Adapting *n* = 191, 55, 15, Adapting *n* = 88, 38, 15, Non-adapting *n* = 27, 3, 31, LME, Depressed *p* = 0.16, 0.34, 0.98, Baseline Adapting *p* = 0.34, 0.020, 0.098, Adapting *p* = 5.0 × 10^−5^, 0.98, 0.0080, Non-adapting *p* = 7.5 × 10^−4^, 8.1 × 10^−9^, 0.49). (e) On Day 0, less adapting neurons are found closer to the electrode (Kruskal–Wallis, *p* = 0.033, 3.6 × 10^−7^, 1.2 × 10^−10^, 8.2 × 10^−17^, 4.1 × 10^−4^). By Day 1, depressed adapting neuron profiles are closer to the electrode while adapting neuron profiles are further away (*p* = 0.004, 0.008, 0.65, 1, 1, 1). On Day 2, there is no significant difference in the spatial distribution of the neuron profiles (*p* = 0.93, 0.92, 0.68, 1, 0.96, 0.97).

Building on the observed changes in microglial behavior and their potential interaction with neuronal activity, we next assessed how neuronal activation profiles vary in relation to the electrode and microglial response. Neurons exhibit different activity profiles during ICMS depending on distance from the electrode [10, 50, 70–72]. A sample of chronic spatial neuron activity revealed a varied and seemingly inconsistent distribution of neuronal activation profiles (figure 6(a)). Four distinct activation profiles emerged when examining the neurons: (1) depressed neurons, which show diminished calcium activity post-activation, (2) baseline adapting neurons, which adapt back to baseline levels during stimulation (3) adapting neurons, which exhibited decreased calcium activity but still above a threshold of one standard deviation, and (4) non-adapting neurons, which demonstrated sustained activation throughout stimulation (figure 6(b)). These neuronal response magnitudes differed across days (LME Days *p*= 2.7 × 10^−19^)

Despite the spatial variability in activation profiles, there were no significant or consistent changes in the proportion of these profiles across the days during low-frequency ICMS (figure 6(c), in order Day 0, Day 1, Day 2: Depressed: 7.1 ± 5.5%, 24.7 ± 18.2%, 6.4 ± 6.4%, Baseline Adapting: 47.9 ± 16.0%, 27.6 ± 11.7%, 21.0 ± 10.6%, Adapting: 33.0 ± 14.4%, 27.6 ± 10.1%, 44.7 ± 20.3%, Non-adapting: 14.3 ± 8.7%, 10.1 ± 10.7%, 20.3 ± 11.8%). However, despite the stable proportions, non-adapting neurons were located significantly farther from the electrode (figure 6(d), in order Day 0, Day 1, Day 2 Depressed: 182.4 ± 16.5 *µ*m, 139.1 ± 13.5 *µ*m, 134.2 ± 29.5 *µ*m, Baseline Adapting: 204.8 ± 6.1 *µ*m, 192.6 ± 10.5 *µ*m, 152.9 ± 20.4 *µ*m, Adapting: 135.5 ± 8.3 *µ*m, 194.1 ± 10.5 *µ*m, 154.0 ± 17.0 *µ*m, Non-adapting: 65.2 ± 7.7 *µ*m, 191.8 ± 12.9 *µ*m, 165.0 ± 13.1 *µ*m). Both adapting (*p* = 5.0 × 10^−5^) and non-adapting (*p* = 7.5 × 10^−4^) neurons shifted significantly away from the electrode by Day 1, which opposes the typical migration of microglia towards an injury site (the electrode).

Interestingly, on Day 0, non-adapting neurons were localized significantly closer to the electrode compared to other profiles (figure 6(e); *p*< 3.6 × 10^−7^), and adapting neurons were also closer to the electrode than depressed neurons (figure 6(e); *p* = 0.033). By Day 1, depressed neurons were located significantly closer to the electrode than baseline (*p* = 0.0040) or adapting neurons (*p* = 0.0080; figure 6(e)), suggesting that the spatial distribution of sustained neuronal activation may shift as part of the chronic injury response. Although neuron recruitment and magnitude of activation is often related to distance from the electrode, the proportion and spatial localization of depressed neurons do not exhibit significant changes across days (supplemental figure 6). While neuron activation seems to be influenced by proximity to the electrode, the lack of change in proportions and distances for depressed neurons, coupled with variability across animals, indicates that additional factors are at play. Microglia–neuron interactions are the most likely candidates for influencing neuronal activation in this context.

**Figure 7.**
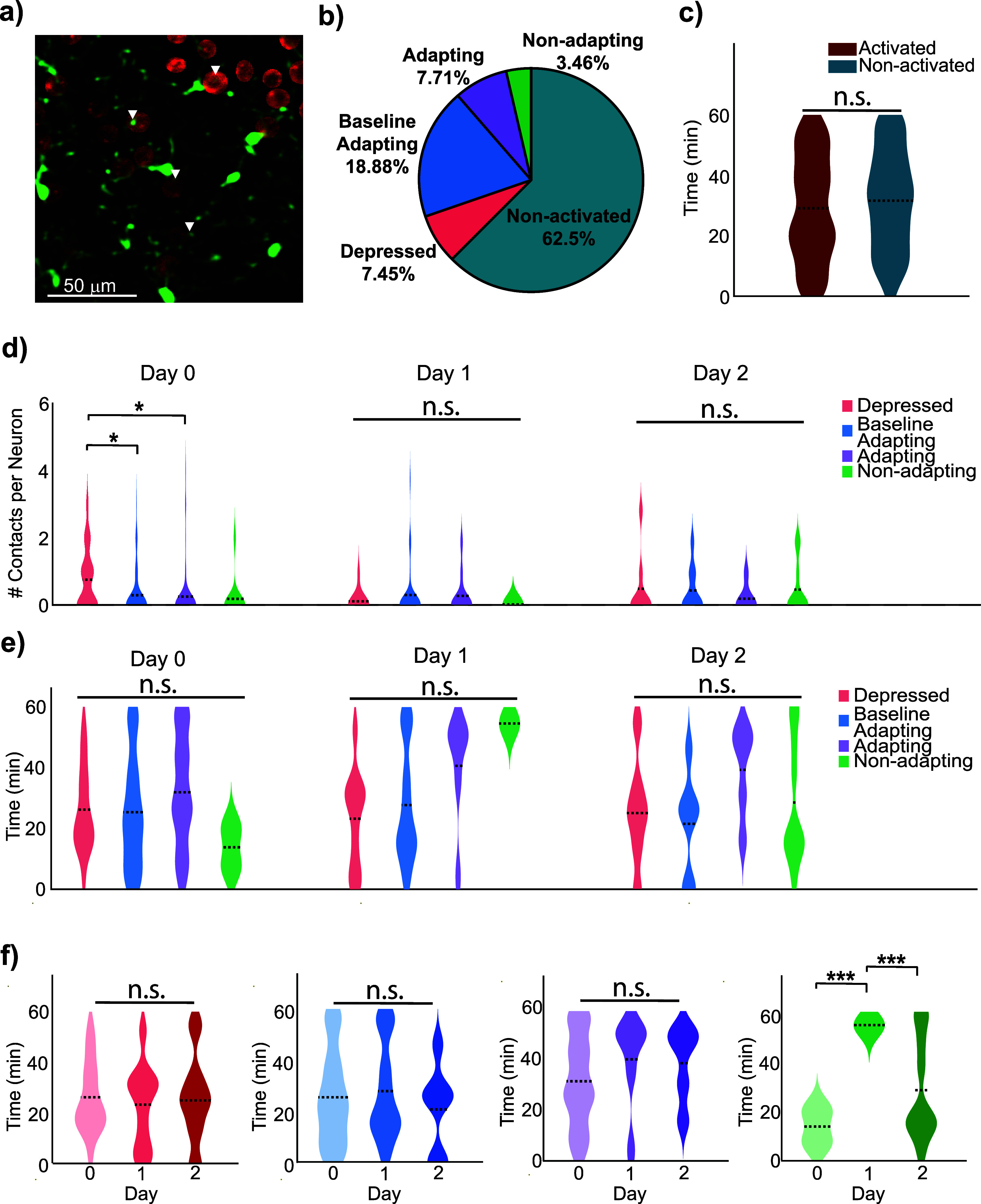
Microglia preferentially contact depressed neurons transiently on Day 0. (a) Two-photon microscopy of microglia in proximity to neuronal somas was used to measure microglia–neuron interactions. (b) Percentage of neuron profiles across all chronic timepoints. (c) Peri-stimulation analysis of the timing of microglia interactions with activated and non-activated neurons, showing no significant difference between the timing of interactions (*N* = 99, 123 neurons, *n* = 45, 83 peri-stimulation interactions, one-way repeated measures ANOVA, *p* = 0.42). (d) Microglia interact more frequently with neurons that demonstrate more suppressed activation profiles on Day 0 (LME main effect of neuronal activation profile *p*= 0.014; Depressed *N* = 20, 20, 8, *n* = 16, 10, 6, peri-stimulation interactions, Baseline Adapting *n* = 124, 36, 9 neurons, Adapting *n* = 56, 22, 11neurons, Non-adapting *n* = 11, 1, 21 neurons, one-way repeated measures ANOVA, Day 0 *p* = 0.046, 0.044, Day 1 *p* = 0.73, Day 2 *p* = 0.75). Per-day significance is reported in the Results; only Day 0 shows a significant within-day preference for depressed neurons, whereas Day 1 and Day 2 do not reach significance. (e) Peri-stimulation timing of microglia interactions with different neuron activation profiles is not significantly different (one-way repeated measures ANOVA, *p* = 0.38, 0.074, 0.43). Violin plots show the distribution of microglial contacts with neuronal somas across the 1 h stimulation period for each activation profile. The width of each violin represents the density of interactions within 1-minute time bins, not absolute counts. This provides a visual representation of when microglia are more or less likely to contact neurons during stimulation, highlighting temporal trends without implying cumulative interaction numbers. (f) Peri-stimulation timing of microglia–neuron interactions separated by identified neuron activation profiles. Overall, microglia interact at significantly different times depending on neuron profile (LME main effect, *p* = 2.2 × 10^−7^). However, pairwise comparisons across days reveal no significant differences in timing for specific neuron profiles (Depressed *N* = 13, 8, 7 neurons, *n* = 16, 10, 6, peri-stimulation interactions, baseline adapting *N* = 51, 15, 5 neurons, *n* = 69, 20, 6, peri-stimulation interactions, adapting *N* = 18, 8, 3 neurons, *n* = 19, 12, 5, peri-stimulation interactions, non-adapting *N* = 1, 1, 11 neurons, *n* = 2, 1, 10, peri-stimulation interactions, LME, *p* = 0.44, 0.63, 0.36, 2.2 × 10^−7^; Day 0 vs Day 1 *p* = 7.8 × 10^−8^, Day 1 vs Day 2 *p* = 2.1 × 10^−7^).

To further investigate the role of microglia in modulating neuronal activity during ICMS, we analyzed the spatial and temporal dynamics of microglia–neuron interactions on a day-specific basis. By isolating neuron somas and identifying when microglia processes made and broke putative contact with nearby neurons (figure 7(a)), we quantified both the timing and frequency of microglia–neuron interactions (figures 7(b) and (c)). On Day 0, microglia interacted significantly more frequently with post-activation depressed neurons than with baseline (*p*= 0.046) and adapting (*p* = 0.044) (figure 7(d), Day 0: depressed: 0.75 ± 0.20, baseline: 0.29 ± 0.06, adapting: 0.25 ± 0.11, and non-adapting: 0.18 ± 0.18 contacts per neuron), suggesting a potential suppressive effect on neuronal activity.

LME analysis confirmed a main effect of neuronal activation profile across all days (*p* = 0.014), indicating that neuronal state influences microglia contact frequency at the population level. However, this effect was not driven by consistent per-day differences, as within-day contrasts on Day 1 and Day 2 did not reveal preferential targeting of depressed neurons. Instead, on Day 1, non-activated neurons received more contacts than depressed neurons (supplemental figure 7(c), *p*= 0.006), indicating a shift toward engagement with neurons operating near baseline activity. Overall, microglia interact at significantly different times depending on neuronal activation profile (LME main effect, *p* = 2.2 × 10^−7^; figure 7(f)), highlighting temporal specificity in these interactions. Together, these results indicate that microglia–neuron contact preferences are dynamic and context-dependent, with preferential targeting of depressed neurons restricted to the acute post-stimulation period.

Across Days 1 and 2, no significant within-day differences in interaction frequency were observed among neuron activation profiles, indicating that these Day 0 effects are transient. Similarly, the timing of microglia–neuron interactions did not significantly differ between activated and non-activated neurons (figure 7(c)), across activation profiles at individual time points (figure 7(e)), or across days (figure 7(f)). While some visual differences were apparent, the limited number of adapting and non-adapting neurons likely reduced statistical power. These results suggest that acute, within-session microglia–neuron interaction timing is largely stable, consistent with prior observations that early post-implantation neuronal activity is transiently altered and partially suppressed by inflammatory processes [62, 65].

**Figure 8.**
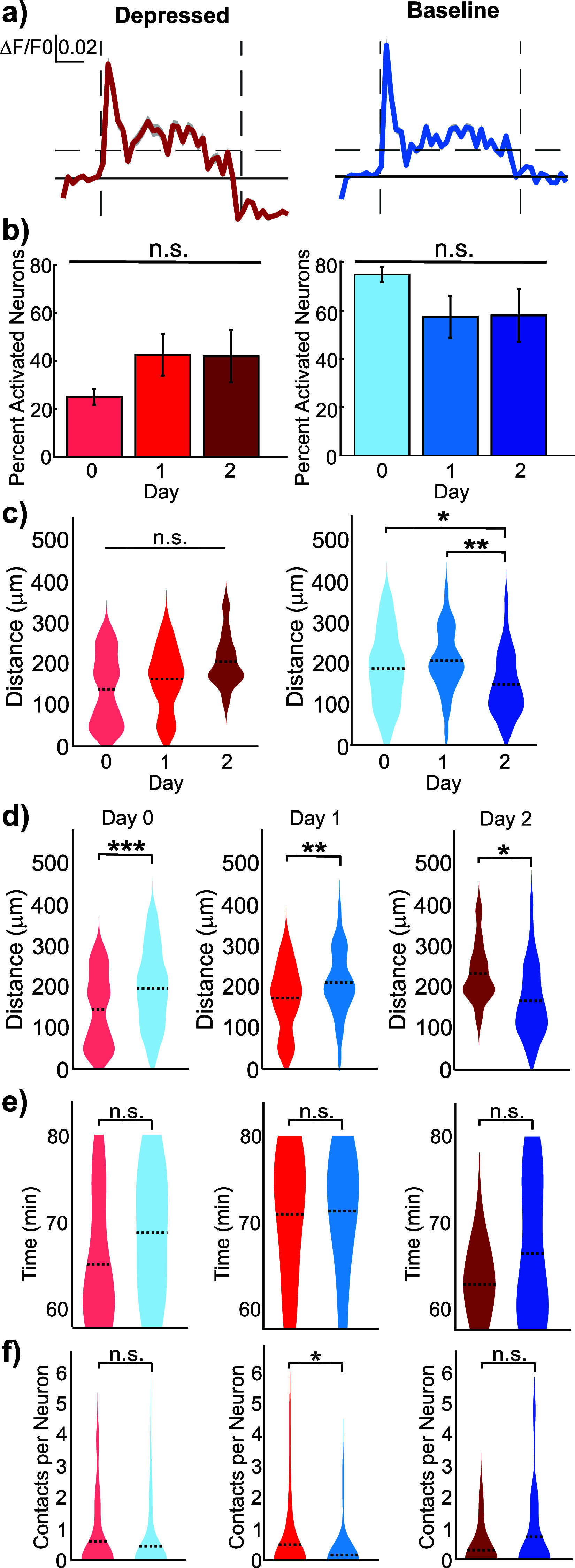
Microglia contacts with post-stimulation neurons are transiently associated with depressed activity. (a) Representative calcium traces of neurons with baseline or depressed activity profiles following stimulation. (b) The proportion neurons exhibiting baseline versus depressed post-stimulation profiles did not significantly change across the days (*N* = 4 mice, LME, *p* = 0.65). (c) Distance changes across days were profile-dependent (LME main effect of Profile, *p* = 3.95 × 10^−3^, LME Day × Profile interaction, *p* = 1.99 × 10^−3^), with depressed post-stimulation activity shifting farther from the electrode across days (n = 263, 60, 72 depressed neurons, LME, *p* = 0.19) while neurons with baseline post-stimulation profiles slightly increased then decreased significantly on Day 2 (*n* = 72, 80, 11 baseline neurons, LME, *p* = 0.018, 0.0060). (d) On Days 0 and 1, depressed post-stimulation neurons were significantly closer to the electrode compared to baseline neurons, but by Day 2, baseline neurons were significantly closer (LME: Day 0 *p*= 8.8 × 10^−3^, Day 1 *p* = 1.6 × 10^−3^, Day 2 *p* = 6.1 × 10^−3^), highlighting a profile-specific spatial relationship relative to electrode proximity. (e) The timing of microglia–neuron interactions post-stimulation did not significantly differ by activation profile on any day (Depressed *N* = 53, 51, 10 neurons, *n* = 10, 6, 1 poststimulation interactions, baseline *N* = 158, 31, 46 neurons, *n* = 32, 6, 10 poststimulation interactions, one-way repeated measures ANOVA, *p* = 0.16, 0.94, 0.69). Violin plots show the distribution of microglial contacts with neuronal somas across the 1 h stimulation period for each activation profile. The width of each violin represents the density of interactions within 1 min time bins, not absolute counts. This provides a visual representation of when microglia are more or less likely to contact neurons during stimulation, highlighting temporal trends without implying cumulative interaction numbers. (f) On Day 1, microglia contacted depressed post-stimulation neurons significantly more frequently than baseline neurons (Kruskal–Wallis, p = 0.040). No significant differences were observed on Days 0 or 2 (one-way repeated measures ANOVA, *p* = 0.33, 0.30). Across all days, there was a significant effect of Day on microglia contact frequency (LME, *p* = 0.047), indicating a temporal modulation of interactions.

Another critical factor of ICMS is neuronal activity following stimulation. At lower frequencies and shorter durations (1–30 s), a larger proportion of neurons typically return to their baseline activity [10, 70, 72]. Alternatively, some neurons exhibit a significantly depressed calcium signal post-stimulation. Both response types were observed (figure 8(a)), and the proportions of baseline and depressed post-stimulation profiles did not significantly change across days (figure 8(b); LME, *p* = 0.65). Spatial analyses revealed that the post-stimulation profile strongly predicted neuron distance from the electrode (LME main effect of Profile, *p* = 3.95 × 10^−3^). Depressed post-stimulation neurons were located further from the electrode, whereas baseline post-stimulation neurons were closer (figure 8(c) in order Day 0, Day 1, Day 2 Depressed: 137.4 ± 10.0 *µ*m, 161.5 ± 8.6 *µ*m, 203.9 ± 19.0 *µ*m, Baseline: 183.3 ± 5.6 *µ*m, 202.6 ± 9.8 *µ*m, 145.2 ± 9.0 *µ*m). This trend was confirmed by direct comparisons, where depressed neurons were significantly closer to the electrode on Days 0 and 1, but significantly farther on Day 2 (figure 8(d); Day 0: *p* = 0.0001; Day 1: *p* = 0.002; Day 2: *p* = 0.018).

In contrast to the spatial changes, the timing of microglia interactions with neuronal somas post-stimulation did not differ significantly across days (figure 8(e)). Interestingly, interaction frequency was profile dependent. On Day 1, microglia contacted depressed post-stimulation neurons significantly more frequently than baseline neurons, while no differences were observed on Days 0 or 2 (figure 8(f); *p* = 0.040; in order Day 0, Day 1, Day 2 Depressed: 0.60 ± 0.16, 0.49 ± 0.15, 0.30 ± 0.21, Baseline: 0.44 ± 0.08, 0.16 ± 0.12, 0.74 ± 0.19 contacts per neuron). Across all days, LME revealed a main effect of neuronal profiles on microglia contacts (*p* = 0.014), confirming that ICMS-induced neuronal activation state drives microglia engagement, with a modest temporal modulation (LME main effect of Day, *p* = 0.047). Together, these findings indicate that the post-stimulation neuronal profile is a key determinant of both spatial positioning relative to the electrode and the frequency of microglia interactions, whereas day primarily modulates these profile-specific effects. Depressed neurons, which experienced reduced post-stimulation activity, were preferentially contacted by microglia at specific time points, suggesting a role for microglia in regulating or restoring neuronal homeostasis following ICMS.

## 4. Discussion

ICMS evokes heterogeneous neuronal responses that evolve over time, reflecting both immediate circuit-level effects and dynamic neuromodulation. This study demonstrates that microglia–neuron interactions are temporally and spatially regulated during and after stimulation, with microglial processes preferentially extending towards neurons exhibiting suppressed or altered activation states. Using *in vivo* two-photon imaging over three consecutive days, we tracked microglial process interactions with neuronal somas and categorized neuronal calcium responses into distinct activation profiles during 10 Hz ICMS. Analyses revealed that while the *timing* of microglia–neuron interactions did not correlate with activation states, the *rate* of interactions was significantly elevated for suppressed neurons during and after stimulation. These putative contact rates were particularly enriched in activated neurons classified as ‘depressed’ based on Δ*F*/*F* reductions and located closer to the electrode (figures 6(d), (e), 7(d) and 8(d)), supporting the hypothesis that microglia prioritize metabolically or synaptically compromised targets even as they migrate to an implant site. These interactions are consistent with activity-dependent microglial engagement. However, the data are observational and do not establish a homeostatic function. Alternative explanations include targeting of neurons with reduced activity due to local injury or transient dysfunction. Together, these findings highlight the importance of considering glial dynamics in the interpretation and optimization of ICMS-based neuromodulation strategies.

### 4.1. Low frequency ICMS does not induce morphological microglia activation

Our study demonstrates that prolonged 1 h 10 Hz ICMS does not induce classical microglial activation, as evidenced by the absence of morphological changes in directionality- and transition- indices (*D*- and *T*-indices; figure 3). Instead, microglial processes dynamically responded to ICMS by extending toward both the stimulating electrode and active neurons (figures 4(a), 5(g) and (h)), with putative contact frequency inversely correlating with neuronal adaptation magnitude (e.g. depressed neurons exhibited more microglial interactions; figure 7(d)). This targeting was selective despite no changes in average *D*- or *T*-indices (figures 3(c) and (d)), suggesting that microglia adapt their motility to local neurochemical cues without undergoing global activation. Notably, this behavior was associated with neuronal activity, indicating that surveilling microglial responses are partially driven by chemical signaling (e.g. ATP, fractalkine) released by neurons, rather than the electric field [14, 24, 27, 40]. This is consistent with established roles for P2Y12 and CX3CR1 receptors in guiding microglial surveillance in response to ATP and fractalkine, respectively, and likely mediates the preferential process extension toward activated neurons observed on Days 1 and 2 (figures 5(g) and (h)).

Furthermore, microglial processes exhibited robust, time-dependent responses to daily 10 Hz stimulation, rapidly extending toward the electrode during stimulation and partially retracting afterward (figures 3(e) and (f)). While the magnitude of these responses varied slightly across days, the overall temporal pattern of engagement was consistent, indicating that repeated ICMS reliably recruits microglial surveillance without inducing runaway or pathological activation. Microglia–neuron interactions were more frequent for some depressed neurons on Day 1 (figure 8(f)), although these observations remain associative. The patterns are compatible with several mechanisms (homeostatic support, injury surveillance, or phagocytic targeting) and cannot be disentangled in the present dataset. This interpretation is further supported by the gradual appearance of less suppressed neurons further from the electrode (figure 6(d)), implying an active compensatory or monitoring role for microglia.

These findings challenge the assumption that electrical stimulation could directly activate microglia and instead highlight their role as adaptive regulators of neuronal networks. Microglia seem to facilitate a balance in neuronal activity, rather than simply inducing neuroinflammation. However, these interactions may be accompanied by subtle transcriptomic remodeling, such as upregulation of anti-inflammatory markers (e.g. Arg1, IL-10) or synapse-modulating factors (e.g. TREM2, IGF1), which were not assessed in this study but should be explored using post hoc RNA-seq or immunostaining of targeted cells. The subtlety of the response also underscores the importance of stimulation frequency in modulating glial engagement. Higher frequencies (e.g. 40–100 Hz) may drive stronger neuronal adaptation (figure 6(b)) [10, 50, 72] and surpass a threshold for microglial reactivity [73]. Parametric variation of frequency and duty cycle could identify thresholds for transitioning between surveillance, gliomodulation, and inflammatory states. Such profiles would inform precision programming of ICMS paradigms that minimize adverse glial responses.

Given that microglial encapsulation of electrodes coincides with progressive neuronal hypoactivity in chronic implants [6, 62, 74], it is possible that prolonged 10 Hz ICMS over days or weeks could elicit delayed glial adaptations, such as process retraction, phagocytic engagement, or altered cytokine release. These adaptations may underlie the gradual decline in stimulation efficacy observed in long-term neuroprosthetic applications [75]. Extending longitudinal two-photon imaging beyond three days (e.g. to Day 7 or Day 14) would help determine whether transient process accelerations (figures 3(e) and (f)) evolve into more stable structural reorganization, such as sheath formation or synaptic pruning.

Future experiments should focus on tracking microglial dynamics in long-term neuroprosthetic applications, paired with high-resolution imaging of sheath formation around electrodes. Such studies will help determine whether low-frequency ICMS accelerates or mitigates inflammatory encapsulation. Additionally, combining chronic ICMS with longitudinal electrophysiology could clarify whether microglial interactions predict late-phase neuronal silencing or aberrant plasticity. For example, spatial remapping of depressed neuron distributions post-stimulation (figures 8(c) and (d)) may correlate with local field potential changes and could be modulated by prior microglial engagement. Integration of ΔF/F imaging with field recordings would offer a multi-modal metric of microglia–neuron feedback. Such studies would bridge the gap between acute microglial surveillance and chronic circuit modulation, informing strategies to sustain stimulation efficacy through glial-targeted interventions.

Ten hertz ICMS does not induce classical microglial activation but modulates their process dynamics in a manner associated with neuronal adaptation. Future studies should systematically explore stimulation parameters and chronic applications to clarify how microglia contribute to circuit responses. Incorporating microglial engagement into stimulation design may improve the stability and precision of neural circuit modulation.

### 4.2. Low frequency ICMS modulates microglia behavior

Although a 1 h, 10 Hz ICMS does not elicit classical morphological activation of microglia (no change in *D*- or *T*-indices; figures 3(c) and (d)), it dynamically modulates microglial behavior in a manner dependent on local circuit activity. During prolonged 10 Hz stimulation, microglial processes extend preferentially toward the electrode shank and along trajectories overlapping regions of heightened neuronal calcium activity (figures 4(a), 5(f)–(h). Microglia made more putative contacts with neurons exhibiting strong adaptation (figure 7(d)), indicating that these interactions are sensitive to changes in neuronal activity rather than reflecting a global activation state [14, 24, 27, 40]. These findings highlight microglia as context-dependent modulators of neuronal responses during ICMS, suggesting that neuron-centric models of neuromodulation may overlook critical contributions from non-neuronal elements.

Daily 1 h, 10 Hz electrical stimulation induces a clear, time-dependent response of microglial processes. Before stimulation, processes are relatively quiescent, performing routine surveillance. Within the first minutes of stimulation, processes rapidly extend, reflecting an active sensing of the local tissue environment. Extension continues into the middle of the stimulation period, reaching a peak, likely driven by neuronal and astrocytic release of signaling molecules such as ATP or other danger-associated molecular patterns, rather than direct electrical depolarization [24, 39–41]. For example, field-evoked glutamate release and downstream ATP hydrolysis to adenosine are known to regulate P2Y12R-A1R pathways, dampening microglial ramification without triggering a full‐scale inflammatory profile [76, 77]. Toward the end of the stimulation, process extension plateaus or partially retracts, suggesting that microglia approach maximal functional engagement or that local homeostatic mechanisms begin to limit further extension. After stimulation ends, processes partially retract, returning toward their baseline surveillance state, indicating that microglia are dynamically adjusting to the changing local environment. Retraction rates are slower than the initial extension, consistent with the idea that withdrawal is a controlled, recovery process rather than an abrupt withdrawal. These observations demonstrate that microglia are active, temporally structured responders, with peak recruitment during sustained stimulation and gradual disengagement afterward (figures 3(e) and (f)).

Although these process dynamics were consistently elicited by electrical stimulation, they occur within a local environment shaped by multiple overlapping cues. Within ∼150 *µ*m of the electrode, both the injury itself and changes in neuronal activity create competing cues that influence how microglial processes ultimately orient. By Day 1, the polarity index for extensions peaked near the electrode, reflecting an injury response (figures 2(d) and (e)). Notably, 10 Hz stimulation did not significantly amplify overall polarization, but instead drove process extensions toward surrounding activated neurons (figures 5(g) and (h)). As neurons near the electrode exhibited more depressed activity over time (figures 6(d) and (e)), microglial processes remained oriented toward the neurons, suggesting a link between sustained low neuronal activity and increased microglial engagement. On Day 1, microglia showed increased engagement with neurons exhibiting depressed post-stimulation Δ*F*/*F* (figure 8(f)), implying that microglia sense and respond to regions of metabolic stress. This aligns with the shift in neuronal activity profiles, where depressed neurons are more frequently contacted by microglia during stimulation (figure 7(d)). The observed temporal pattern aligns with prior observations that, as neurons near an active electrode experience metabolic strain [78], they exhibit decreased BOLD-OIS amplitudes and eventual silencing [31]. Microglia likely detect local shifts in extracellular adenosine or other metabolic byproducts and extend processes to these regions of metabolic stress. During stimulation, microglia may aid in clearing extracellular metabolites, modulating extracellular ion balance, or releasing trophic factors to restore homeostasis, actions analogous to their roles in injury repair and synaptic refinement [16, 37]. As metabolic stress increase near implants [31, 78, 79], microglial engagement may help stabilize network function without inducing pro-inflammatory phenotypes. These dynamics highlight how microglia respond flexibly to both structural and functional cues in the tissue, linking general motility to subsequent, activity-dependent engagement during ICMS.

The progressive shift of non-adapting neurons away from the electrode (figure 6(d)), combined with microglial extensions toward the electrode (figures 2(d) and (e)) suggests that low-frequency ICMS fosters a dynamic equilibrium: microglia monitor metabolic stress and help preserve tissue homeostasis without driving overt gliosis. This interpretation is supported by studies demonstrating that 10 Hz ICMS suppresses pericyte calcium activity in a frequency-dependent manner, suggesting coordinated neuromodulation of non-neuronal cells [47]. Such responses are unlikely to arise from direct depolarization, further supported by the fact that the direct activation volume appears limited ∼10–20 *µ*m from the electrode site as measured by iGluSnFR [80]. Instead, the collective findings implicate purinergic or adenosine-mediated signaling cascades play a significant role in tissue responses to ICMS [24, 41, 81, 82]. Therefore, neuromodulation protocols may benefit from accounting for microglial dynamics by tailoring ICMS parameters (frequency, duty cycle, amplitude) to align with microglial surveillance windows to stabilize tissue responses and prolong functional integration. Incorporating biomaterials engineered to optimize charge transfer and modulate microglial membrane potentials, such as conductive polymer coatings that limit ATP release, may further harness these glial mechanisms to support biocompatible long-term implants.

Our study highlights the layered, dynamic nature of microglia–neuron interactions during ICMS. Continuous metrics of neuronal activation, such as area under the calcium response curve, were explored but limited by imaging heterogeneities and tracking challenges. We therefore focused on discrete response profiles, which are more robust across animals. These findings provide a framework for understanding microglia–neuron interactions during ICMS and will guide future studies with additional animals to explore continuous correlation analyses and further assess microglia’s modulatory role.

### 4.3. Microglia–neuron interactions

Our data indicate that during 10 Hz ICMS microglial processes transiently and preferentially orient toward neurons exhibiting altered activity (figures 4(a), 5(g) and (h)). This transient positional association evolves over space and time, aligning with shifts in neuronal stress, rather than representing a static or cross-day effect. Day serves as context for this dynamic system, not as an outcome. These observations suggest that microglia are not merely reacting to electrical stimulation driven neuronal activity, but are actively adjusting their targeting based on rapidly changing local neuronal states, proximity to injury, and local inflammatory signals.

Although this stimulation paradigm does not induce classical inflammatory activation in surveilling microglia (no change in *D*- or *T*-indices; figures 3(c) and (d)), microglial processes nevertheless exhibit highly dynamic directional plasticity, suggesting a role in fine‐tuning synaptic function rather than merely reacting to injury. Microglia express diverse neurotransmitter receptors and voltage‐sensitive receptors [6, 25, 83, 84], enabling direct sensitivity to ICMS-evoked glutamate or GABA release. Neuronal activity under 10 Hz ICMS may lead to the release of several neurochemicals that likely guide microglial behavior. Elevated extracellular ATP released by hyperactive neurons engages microglial P2Y12 receptors, promoting rapid process extension toward active or stressed synapses [76, 77]. ATP hydrolysis to adenosine via microglial CD39/CD73 triggers A1 receptor signaling on neurons, temporarily dampening excitability and providing negative feedback that restrains microglial over-engagement [76, 85]. Likewise, fractalkine (CX3CL1) released from stimulated neurons binds microglial CX3CR1, regulating chemotaxis and synaptic pruning [86–88]. It is important to note that our animal model was heterozygous for CX3CR1, preserving one functional allele. Excess glutamate, abundant during sustained activation, can engage microglial mGluRs and, if unregulated, drive pro-inflammatory signaling [89, 90]. Together, these signals coordinate microglial process extension and retraction in a temporally precise manner to maintain network stability.

While neuron activation is influenced by proximity to the electrode, variability in the spatial distribution of depressed neurons across animals highlights the transient and context-dependent nature of local neuronal stress, suggesting additional modulatory factors such as astrocyte activity, interneuron dynamics, vascular perfusion, or subtle tissue damage. Microglia–neuron interactions emerge as the most likely contributors to neuronal depression because spatially and temporally coordinated contacts correlate with transiently depressed neuronal activation patterns (figure 7(d)). As expected, microglial interaction frequency is highest for neurons exhibiting the greatest adaptation [46, 78]. For example, on Day 1, a temporary spike in microglial contacts aligns with a local cluster of depressed neurons near the electrode, demonstrating that microglial engagement is adaptive and rapidly responsive to both metabolic state and proximity to injury. Depressed or highly adapting neurons consistently receive more frequent, yet transient, microglial putative contacts (figure 7(d)), reinforcing that this is a highly dynamic, physiologically meaningful process, not a statistical artifact.

Physical microglia–neuron interactions during ICMS are likely guided by local concentrations of ATP, adenosine, and fractalkine. ATP and fractalkine drive targeting toward regions of high neuronal firing, while adenosine signals to restrain engagement once excitability subsides [21, 76, 77]. Additionally, low-frequency ICMS (2–20 Hz) enhances release of BDNF and other trophic factors, suppressing pro-inflammatory cytokine production and promoting angiogenesis [42, 43, 91]. By contrast, higher frequencies (e.g. 40 Hz) induce robust microglial gene expression and morphological changes [73, 92]. Although we did not observe morphological activation at 10 Hz (figures 3(c) and (d)), this frequency-dependent balance between trophic and inflammatory signaling shapes the extent, directionality, and temporal transience of microglia–neuron interactions observed during ICMS (figures 5(f)–(h) and 7(d)).

However, these observations remain correlative. To establish causality, future experiments should manipulate specific signaling pathways (e.g., P2Y12 or CX3CR1) to determine whether disrupting ATP/P2Y12 or fractalkine signaling decouples microglial process extension from neuronal activity during ICMS [81, 93]. Similarly, CX3CR1 knockout models could determine whether fractalkine-mediated chemotaxis underlies process polarization toward active neurons [82]. Such manipulations would reveal whether physical microglia–neuron interactions directly contribute to ICMS-induced neuronal adaptation or merely reflect underlying metabolic or physiological stress. Investigating whether repeated, chronic 10 Hz ICMS maintains this highly adaptive interplay or eventually drives maladaptive microglial states will be critical for designing stimulation protocols that sustain beneficial microglial engagement while avoiding deleterious inflammation in chronic implant settings.

## 5. Limitations

While 10 Hz ICMS directed microglial processes toward active neurons (figures 5(f)–(h)) and contact frequency correlated with neuronal adaptation (figure 7(f) and 8(f)), several caveats apply. First, we analyzed only soma contacts, omitting interactions with dendrites, axons, or synapses, where microglia prune weak connections and influence neurotransmission [45, 94, 95]. Second, the 1 h ICMS window captures only acute responses to stimulation (figures 3(c)–(f)). Chronic stimulation may induce delayed glial adaptations, such as phagocytosis of stressed neurons or process retraction, and shifts in neuronal profiles (figures 6(d) and (e)), which require longitudinal imaging. Third, examining only 10 Hz limits the understanding of how microglia respond to other frequencies and pulse parameters. Higher frequencies (40–100 Hz) can induce pro-inflammatory microglial changes [73, 92], while kilohertz-range waveforms may suppress reactivity. A systematic exploration of frequency, charge density, and waveform shape is needed to identify regimes that avoid unintended activation. Fourth, although ATP/P2Y12 and fractalkine/CX3CR1 signaling likely mediate process extension toward active neurons [21, 76, 77, 86], we did not directly test these pathways. Blocking P2Y12 or CX3CR1 could reveal whether microglial motility requires those signals. Nonetheless, our findings indicate that microglia preferentially target neurons with suppressed or adapting responses, suggesting that neuron-derived cues [45] such as reduced activity-dependent ATP or chemokine release, rather than nonspecific electric field effects, drive this behavior. Furthermore, the electric field produced by ICMS decays rapidly with distance, influencing tissue within a range of ∼10 *µ*ms [80], making it unlikely that microglia–neuron interactions beyond this radius are directly modulated by the field itself. We also did not measure microglial Ca^2+^ transients, which drive process movement [84], so it remains unclear if ICMS directly triggers intracellular signaling. Finally, baseline jRgeco1a fluorescence varied between neurons, but all neurons classified as ‘non-responsive’ had detectable resting fluorescence with minimal Δ*F*/*F* changes post-ICMS. While it is true that neurons with sub-threshold jRgeco1a expression or low basal activity could be missed, the spontaneous activity seen in these same fields during pre-stimulation imaging provides further evidence that such neurons were functionally silent during the stimulation window. Future studies should address these gaps to clarify how ICMS shapes microglial contributions to circuit modulation and combine per-neuron activity analyses, targeted pathway perturbations, and longitudinal imaging to determine whether microglial contacts reflect homeostatic support, injury surveillance, or removal of compromised neurons.

## 6. Conclusion

This study demonstrates that low-frequency (10 Hz) ICMS does not provoke classical microglial activation but appears to engage microglia in a manner associated with neuronal activity states. Through real-time imaging, we revealed that microglial processes dynamically interact with neurons during stimulation, with putative contact more frequent for neurons exhibiting reduced or absent activation during stimulation, and in some conditions were inversely related to the magnitude of the neuronal adaptation. These findings indicate that microglial process behavior is associated with stimulation-altered neuronal activity. They do not exclude concurrent effects of injury, metabolic stress, or local microenvironmental changes. Crucially, the present data do not establish a causal or exclusively homeostatic role for microglia in regulating neuronal excitability. Instead, the observed contact patterns are consistent with microglia responding to neurons exhibiting altered or suppressed activity states, which may reflect local injury, functional stress, or transient dysfunction during ICMS. The fact that several effects were most prominent on a single post-implantation day further suggests that microglial engagement is context-dependent and shaped by the acute tissue environment rather than representing a uniform stabilizing mechanism.

These results nonetheless highlight microglia as active participants in the acute neural response to intracortical stimulation, especially via process movement. By demonstrating that microglial-neuronal interactions are modulated by stimulation-altered neuronal activity, this work motivates future studies that explicitly test whether microglia preferentially target neurons with reduced firing or compromised function, and whether such interactions influence subsequent neuronal recovery or adaptation. Longitudinal experiments combined with targeted perturbations of microglia–neuron signaling will be essential to determine whether these interactions contribute to circuit stabilization, injury surveillance, or other adaptive processes during neurostimulation. Incorporating such a glia-aware framework may ultimately improve the interpretability and long-term efficacy of neurostimulation technologies by accounting for non-neuronal contributions to circuit-level responses.

## Data Availability

The raw/processed data required to reproduce these findings cannot be shared at this time as the data also forms part of an ongoing study. The data that support the findings of this study are openly available at the following. URL/DOI: www.bioniclab.org/links [96]. Supplementary Material available at https://doi.org/10.1088/1741-2552/ae4652/data1.

## Availability of data and materials

The datasets generated and used during the current study are available from the corresponding author on reasonable request.
